# Optimal policy for value-based decision-making

**DOI:** 10.1038/ncomms12400

**Published:** 2016-08-18

**Authors:** Satohiro Tajima, Jan Drugowitsch, Alexandre Pouget

**Affiliations:** 1 Département des Neurosciences Fondamentales, University of Geneva, Rue Michel-Servet 1, Genève 1211, Switzerland; 2 Department of Neurobiology, Harvard Medical School, 220 Longwood Avenue, Boston, Massachusetts 02115, USA; 3 Department of Brain and Cognitive Sciences, University of Rochester, Rochester, NY USA; 4 Gatsby Computational Neuroscience Unit, University College of London, London, UK

## Abstract

For decades now, normative theories of perceptual decisions, and their implementation as drift diffusion models, have driven and significantly improved our understanding of human and animal behaviour and the underlying neural processes. While similar processes seem to govern value-based decisions, we still lack the theoretical understanding of why this ought to be the case. Here, we show that, similar to perceptual decisions, drift diffusion models implement the optimal strategy for value-based decisions. Such optimal decisions require the models' decision boundaries to collapse over time, and to depend on the *a priori* knowledge about reward contingencies. Diffusion models only implement the optimal strategy under specific task assumptions, and cease to be optimal once we start relaxing these assumptions, by, for example, using non-linear utility functions. Our findings thus provide the much-needed theory for value-based decisions, explain the apparent similarity to perceptual decisions, and predict conditions under which this similarity should break down.

## Abstract (web summary)

Drift diffusion models (DDM) are fundamental to our understanding of perceptual decision-making. Here, the authors show that DDM can implement optimal choice strategies in value-based decisions but require sufficient knowledge of reward contingencies and collapsing decision boundaries with time.

## Graphical abstract

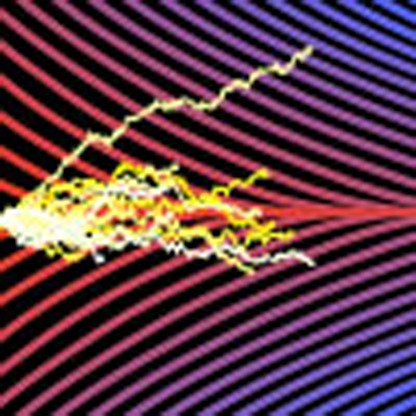

In everyday ambiguous and noisy environments, decision-making requires the accumulation of evidence over time. In perceptual decision-making tasks (for example, discriminating a motion direction), choices and reaction times are well-fit by drift diffusion models (DDMs)^1^,^2^,^3^. These models represent the accumulated belief informed by sensory evidence as the location of a diffusing particle that triggers a decision once it reaches one of two decision boundaries. DDMs are known to implement theoretically optimal algorithms, such as the sequential likelihood ratio test^4^,^5^,^6^ and more general algorithms that handle varying task difficulty^7^.

Recently, DDMs have been shown to also describe human behaviour in value-based decisions, where subjects compare the endogenous values of rewarding items (for example, deciding between two lunch options). This suggests that humans perform value-based decisions by computations similar to those used for standard perceptual decision (such as visual discrimination of random dot motion directions). In this case, the DDMs are driven only by the difference in item values, and thus predict the choices to be insensitive to the absolute values of the compared items (Fig. 1)^8^,^9^,^10^. In particular, relying only on the relative value means that it might take on average longer to decide between two equally good options than between items of very different values.

This raises an important question: do DDMs indeed implement the optimal strategy for value-based decisions? Intuitively, absolute values should also influence the decision strategy, such that relying only on relative values appears suboptimal. In particular, it seems unreasonable to wait for a long time to decide between two nearly similar highly rewarding options. Nonetheless, DDMs or related models are generally better at explaining human behaviour than alternative models. For example, race models (RMs) assume independent ‘races' to accumulate evidence for individual options. Once one of these races reaches a decision criterion the corresponding choice is triggered^11^,^12^. Even though RMs are sensitive to absolute choice values and as such predict more rapid choices for higher rewarded options, they neither fit well human behaviour in perceptual decision-making tasks^12^ nor in value-based decision tasks in which decisions are usually better described by relying only on relative values^13^,^14^. Does this mean that humans use DDMs even though these model implement suboptimal strategies, or that DDMs indeed implement the optimal strategy for value-based choices? What is clear is that we need to understand (i) what the optimal strategy for value-based decisions is, (ii) why the value-based and perceptual decision seem to be fitted by the same class of models (DDMs) despite the qualitative difference between these tasks and (iii) to which degree value-based and the perceptual decisions differ in terms of their normative computational strategies.

In this paper, we derive the theoretically optimal strategy for value-based decisions, and show that this strategy is in fact equivalent to a particular class of DDMs that feature ‘collapsing boundaries' whose distance shrinks over time. We show that the exact shape of these boundaries and the associated average reaction times depend on average-reward magnitudes even if decisions within individual trials are only guided by the relative reward between choice options. Finally, we highlight the difference between value-based and standard perceptual decisions, reveal specific conditions under which the optimality of DDMs are violated, and show how to reconcile the ongoing debate on whether decision makers are indeed using collapsing decision boundaries. In contrast to previous work that assumed particular *a priori* mechanisms underlying value-based choices, such as RMs or DDMs^15^,^16^,^17^,^18^, our work instead deduces optimal decision-making mechanisms based solely on a description of the information available to the decision maker. Thus, the use of diffusion models for value-based choices is not an *a priori* assumption of our work, but rather a result that follows from the normative decision-making strategy.

## Results

### Problem setup and aim

Consider a decision maker choosing between options that yield potentially different rewards (or ‘values'), as, for example, choosing between two lunch menu options in the local restaurant. If the decision maker knew these rewards precisely and immediately then she should instantly choose the more rewarding option. However, in realistic scenarios, the reward associated with either option is uncertain *a priori*. This uncertainty might, for example, arise if she has *a priori* limited information about the choice options. Then, it is better to gather more evidence about the reward associated with the compared options before committing to a choice (for example, when choosing among lunch menus, we can reduce uncertainty about the value of either menu by contemplating the composition of each menu course separately and how these separate courses complement each other). However, how much evidence should we accumulate before committing to a choice? Too little evidence might result in the choice of the lower-rewarding option (the less appreciated lunch menu), whereas long evidence accumulation comes at the cost of both time and effort (for example, missing the passing waiter yet another time). In what follows, we formalize how to best tradeoff speed and accuracy of such choices, and then derive how the decision maker ought to behave in such scenarios. We first introduce each component of the decision-making task in its most basic form, and discuss generalizations thereof in later sections.

We assume that, at the beginning of each trial, the two options have associated true rewards, *z*_1_ and *z*_2_, which are each stochastically drawn from separate normal distributions with a fixed mean 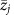 for option *j*∈{1,2} and common variance 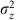. These true rewards are unknown to the decision maker, as they are never observed directly. Instead, we assume that the decision maker observes some momentary evidence with mean 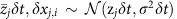 for both options *j*∈{1,2} simultaneously in small time-steps *i* of duration *δt*. Note that variability (and associated ambiguity) of the momentary evidence can arise through noise sources that are both internal or external to the decision maker—sources that we discuss in more detail further below.

**Figure 2:**
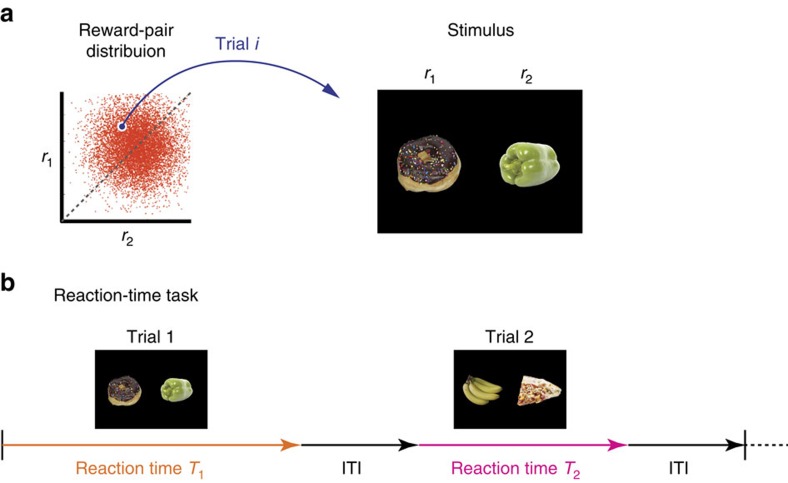
Typical value-based decision-making task. (**a**) (left) Prior distribution from which the rewards for a pair of options are sampled. *r*_1_ and *r*_2_ indicate the reward magnitudes for individual options (for example, objects presented on left- and right-hand side of the screen in each trial). (right) A typical visual stimulus in each trial. (**b**) Reaction-time task. In each trial, the decision maker is presented with a pair of options. The decision maker reports her choice as soon as she has decided which of the two options she prefers. The reaction time (*T*_*i*_) can vary among trials, and individual trials are separated by a fixed inter-trial interval.

Before observing any momentary evidence, we assume that the decision maker holds a normally distributed belief 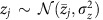 with mean 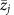 and variance 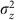, which are, respectively, the mean and variance of the distribution from which the reward are being drawn from at the beginning of each trial. In other words, this a priori belief corresponds to the actual distribution from which the true rewards are drawn (that is, the decision maker uses the correct generative model), and entails that option *j* is most likely to yield reward 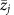, but might also yield other rewards, with the spread of rewards around 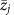 controlled by the level of uncertainty 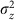 about *z*_*j*_. For now, we only consider the case in which the amounts of reward associated with both options are uncorrelated and, on average, the same 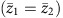. In terms of choosing between lunch menu options, either menu would *a priori* yield the same reward, and the true rewards of either menu option are independently of each other drawn from the aforementioned normal distribution (Fig. 2a). Later, we discuss the consequences of a correlation between true option values.

As soon as being presented with sensory evidence *δx*_*j,i*_, the decision maker accumulates further information about the rewards associated with either choice option. This momentary evidence *δx*_*j,i*_ reveals noisy information about the true reward *z*_*j*_, such that each additional piece of momentary evidence reduces the uncertainty about this reward. We emphasize that neither of the true rewards is ever observed without noise. As a result, the decision maker needs to accumulate evidence to reduce uncertainty about the underlying true rewards by averaging out the noise. Longer evidence accumulation results in a better average and lower associated uncertainty.

The noise in the momentary evidence itself can have both internal and external sources. External sources constitute the potentially stochastic nature of stimuli, perceptual noise, ambiguity and incomplete knowledge. For example, having not yet read the main course and dessert of a particular menu option causes uncertainty about the option's value due to incomplete knowledge. Internal sources could result from uncertain memory, or value inference that extends over time. One example for such value inference would be to sequentially contemplate the value of different features of a particular menu course over time.

Formally, after observing the value-related evidence *δx*_*j*_(0:*t*) from time 0 (onset of momentary evidence) to some time *t*, the decision-maker's posterior belief about the true reward, *z*_*j*_, of option *j* is given by


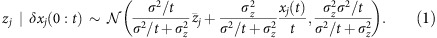


The posterior mean is an evidence-weighted combination of the *a priori* mean 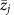
and the time-averaged accumulated evidence 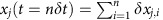, and the posterior variance (that is uncertainty) decreases monotonically with time (see Methods section). Due to uncertainty in the momentary evidence, the accumulated evidence *x*_*j*_(*t*) itself describes a stochastic process. Here, and in contrast to other models of decision-making (both perceptual^19^,^20^ and value-based^15^,^16^), all stochasticity in the accumulated evidence results from ambiguity in the momentary evidence itself, rather than from noise in the mechanisms that implement the decision-making process. In other words, the process responsible for the accumulation of the evidence is assumed to be noiseless, an assumption consistent with recent neurophysiological recordings.^21^

What are the costs and rewards that the decision maker incurs during the course of her decisions? In terms of costs we assume that the decision maker pays a cost *c* per second of accumulating evidence, from onset of the choice options until an option is chosen. This cost could, for example, be an explicit cost for delayed choices, or represent the effort induced by evidence accumulation. In the context of choosing between lunch menus, this cost might arise from missing the passing waiter yet again, or from being late for a post-lunch meeting. Choosing option *j* is associated with experiencing some reward *r*_*j*_ that is a function of the true reward *z*_*j*_ associated with this option, as, for example, when experiencing reward for consuming the lunch. For now, we assume experienced and true reward to be equivalent, that is *r*_*j*_=*z*_*j*_. For a single choice, the overall aim of the decision maker is to maximize expected reward minus expected cost,


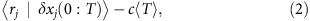


where the expectation is across choices *j* and evidence accumulation times *T*, given the flow of evidence *δx*_*j*_ (0:*T*) from time 0 to *T*. We first derive the optimal behaviour, or ‘policy', that maximizes this objective function for single, isolated choices and later generalize it to the more realistic scenario in which the total reward in a long consecutive sequence is maximized.

### Optimal decisions with DDMs with collapsing boundaries

To find the optimal policy, we borrow tools from dynamic programming (DP). One of these tools is the ‘value function', which can be defined recursively through Bellman's equation. In what follows, we show that the optimal policy resulting from this value function is described by two time-dependent parallel bounds in the two-dimensional space of current estimates of the true option rewards. These bounds are parallel with unity slopes, approach each other over time and together form a bound on the difference of reward estimates. This difference is efficiently inferred by diffusion models, such that DDMs can implement the optimal strategy for value-based decision-making.

**Figure 3:**
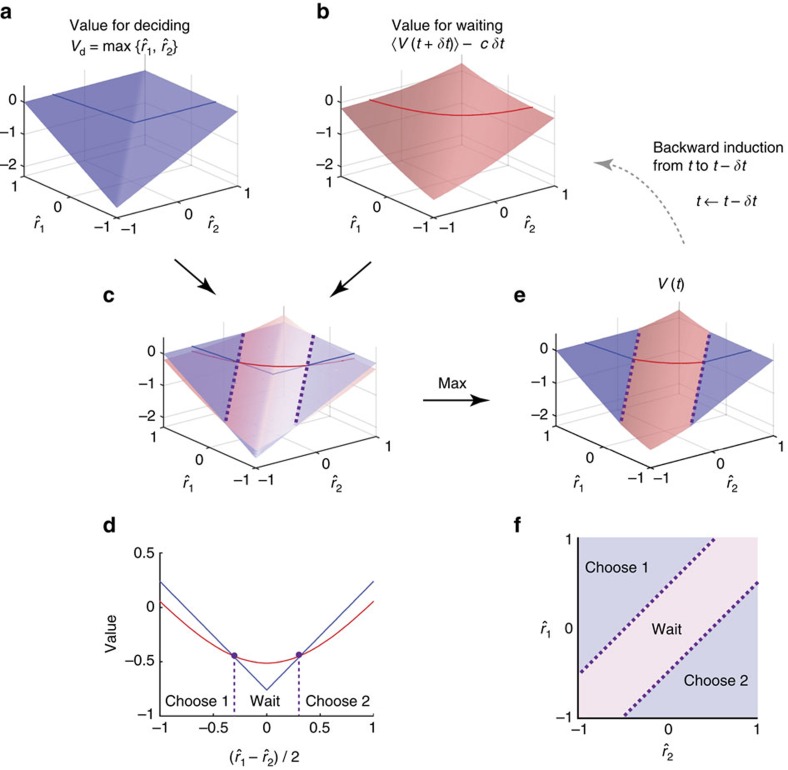
Finding the decision boundaries in value-based decision. (**a**) The expected values of choosing either option are defined as a two-dimensional function (surface), 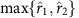, of a pair of reward estimates, 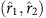, at time *t*. The dark coloured line shows the section at 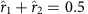. (**b**) Similarly, the value surface for ‘waiting' (that is, the expected value after observing new evidence for a short period *δt*, subtracted cost for waiting *cδt*) is defined as a function of 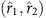. Note that, around the diagonal, 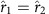, the value for waiting is smoother than that for choosing due to the uncertainty about future evidence. (**c**,**d**) The value surfaces for choosing and waiting superimposed, and their sections at 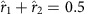. The decision boundaries (dotted lines) are determined by points in the space of reward estimates in which the value for ‘deciding' (blue) equals that for waiting (red). In the region where waiting has a higher value than choosing either option (blue below red curve/surface), the decision maker postpones the decision to accumulate more evidence; otherwise, she chooses the option that is expected to give the higher reward. Because the relationship between the two value surfaces is translational symmetric in terms of mean reward 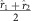, their intersections are parallel and do not depend on this mean reward. (**e**) The expected value *V*(*t*) is given by the maximum of the values for choosing and waiting. This surface determines the value for waiting (**b**) at the next-earlier time step, *t*−*δt*. (**f**) Decision boundaries and associated choices shown in the two-dimensional 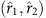
representation. Note that the two boundaries are always parallel to the diagonal, 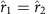. This is because the both value functions (for deciding and for waiting) are linearly increasing with slope one in lines parallel to the diagonal (**a**,**b**). For the value for deciding, for example, below the diagonal we have 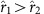, such that 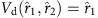, and therefore 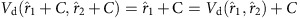, where *C* is an arbitrary scalar. The value for waiting can be shown to have the same property.

*Bellman's equation for optimal value-based decision-making*. To define the value function, assume that the decision maker has accumulated some evidence about the option rewards for some time *t*. Given this accumulated evidence, the value function returns the total reward the decision maker expects to receive when following the optimal policy. This value includes both the cost for evidence accumulation from time *t* onwards and the reward resulting from the final choice. The expected rewards, 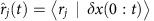, and elapsed time *t* are sufficient statistics of the accumulated evidence (see Methods section), such that the value function is defined over these quantities. At each point in time *t* during evidence accumulation we can either commit to a choice or accumulate more evidence and choose later. When committing to a choice, it is best to choose the option associated with the higher expected reward, such that the total expected reward 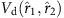 for choosing immediately is given by the value for ‘deciding', 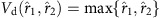 (Fig. 3a). When accumulating more evidence for a small duration *δt*, in contrast, the decision maker observes additional evidence on which she updates her belief about the true rewards while paying accumulation cost *cδt*. At this stage, she expects to receive a total reward of 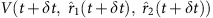. Therefore, the total expected reward for accumulating more evidence is given by the value for ‘waiting', 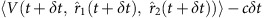 (Fig. 3b), where the expectation is over the distribution of future expected rewards, 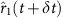 and 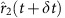, given that they are 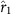 and 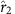 at time *t* (see Methods section for an expression of this distribution). The decision maker ought to only accumulate more evidence if doing so promises more total reward, such that the value function can be written recursively in a form called Bellman's equation (Fig. 3a-c,e; see Supplementary Note 1 for formal derivation),


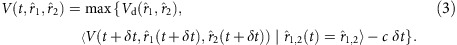


With knowledge of the value function, optimal choices are performed as follows. Before having accumulated any evidence, the subjective expected reward associated with option *j* equals the mean of the prior belief, 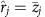, such that the total expected reward at this point is given by 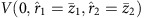. Once evidence is accumulated, 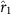 and 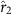 evolve over time, reflecting the accumulated evidence and associated updated belief of the true reward of the choice options. It remains advantageous to accumulate evidence as long as the total expected reward for doing so is larger than that for deciding immediately. As soon as deciding and waiting become equally valuable, that is, 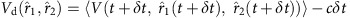, it is best to choose option *j* associated with the higher rewarded expected rewarded 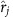. This optimal policy results in two decision boundaries in 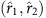-space that might change with time (Fig. 3f). In-between these boundaries it remains advantageous to accumulate more evidence, but as soon as either boundary is reached, the associated option ought to be chosen.

*Parallel optimal decision boundaries*. For the task setup considered above, the decision boundaries take a surprisingly simple shape. When plotted in the 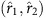-space of estimated option rewards for some fixed time *t*, the two boundaries are always parallel to the diagonal 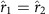 (Fig. 3f). Furthermore, they are always above and below this diagonal, reflecting that the diagonal separates the regions in which the choice of either option promises more reward. Here, we provide an informal argument why this is the case.

The argument relies on the fact that, for each time *t*, the decision boundaries are determined by the intersection between the value for deciding and that for waiting (Fig. 3c,d). Both of these values share the property that, in lines parallel to the diagonal, they are linearly increasing with slope one. Formally, both functions satisfy 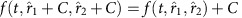 for any fixed time *t*, reward estimates 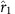 and 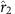, and arbitrary scalar *C*. This implies that, if they intersect at some point 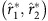, thus forming part of the decision boundary, they will intersect at the whole line 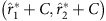 that is parallel to the diagonal (Fig. 3c,e,f). Therefore both decision boundaries are parallel to the diagonal.

How can we guarantee that the values for both deciding and waiting are linearly increasing in lines parallel to the diagonal? For the value for deciding, 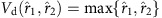, this is immediately obvious from its definition (Fig. 3a and caption). Showing the same for the value for waiting requires more work, and is done by a backwards induction argument in time (see Methods section for details). Intuitively, after having accumulated evidence about reward for a long time (*t*→*∞*), the decision maker expects to gain little further insight by any additional evidence. Therefore, deciding is better than waiting, such that the value function will be that for deciding, 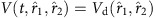, which, as previously mentioned, is linearly increasing in lines parallel to the diagonal, providing the base case. Next, it can be shown that, if the value function at time *t*+*δt* is linearly increasing in lines parallel to the diagonal, then so is the value of waiting at time *t*, and, as a consequence, also the value function at time *t*—essentially because the uncertainty about how the reward estimate evolves over time is shift-invariant (does not depend on current expected rewards, 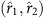; see Methods section). The value function at time *t* is the maximum over the value for deciding and that for waiting. As both increase linearly in lines parallel to the diagonal, so does this value function, 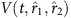 (Fig. 3c,e). This completes the inductive step.

To summarize, an induction argument backward in time shows that both the values for deciding and waiting increase linearly in lines parallel to the diagonal for all *t*. As a consequence, the decision boundaries, which lie on the intersection between these two values, are parallel to this diagonal for all times *t*. In Supplementary Methods, we demonstrate the same property with an argument that does not rely on induction. In both cases, the argument requires, for any fixed *t*, a stochastic temporal evolution of our expected reward estimates that is shift-invariant with respect to our current estimates 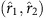. In other words, for any estimates 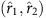, the decision maker expects them to evolve in exactly the same way. This property holds for the task setup described above and some generalizations thereof (Supplementary Note 1), but might be violated under certain, more complex scenarios, as described further below.

**Figure 4:**
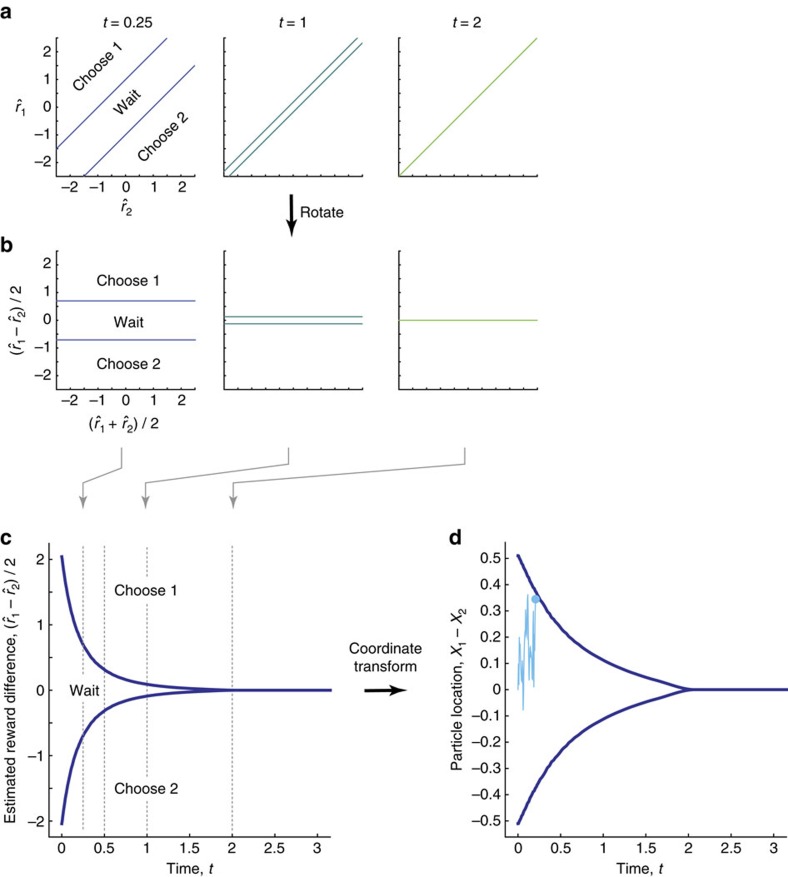
Optimal decision boundaries computed numerically by DP. (**a**) The decision boundaries and choices at three representative time points (*t*=0.5, 1 and 2 s), shown in the space of reward estimates 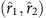. (**b**) The same as **a**, but shown in a rotated space, 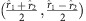. (**c**) The decision boundaries in terms of the expected reward difference, 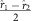, as functions of time. The distance between boundaries decreases as time elapses, and collapses to zero at some point. (**d**) The same decision boundaries shown in the space of accumulated evidence, which is represented by the particle location in a DDM. The cyan trace is a sample particle trajectory, representing evidence accumulation and the subsequent choice of option 1.

*Optimal decisions with collapsing boundaries, and by diffusion models*. A consequence of parallel decision boundaries is that optimal choices can be performed by tracking only the difference in expected option rewards, 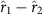, rather than both 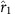 and 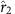 independently. To see this, consider rotating these boundaries in 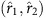-space by −45° such that they come to be parallel to the horizontal axis in the new 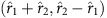-space (Fig. 4a,b). After the rotation they bound 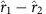 and are independent of 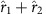.

For Gaussian *a priori* rewards (Fig. 2a), numerical solutions reveal that the distance between the two boundaries decreases over time, resulting in ‘collapsing boundaries' (Fig. 4c) that can be explained as follows. In the beginning of the decision, the true option rewards are highly uncertain due to a lack of information. Hence, every small piece of additional evidence will make the running reward estimates substantially more certain. This makes it worth to withhold decisions by far-separated decision boundaries (Fig. 4c for small *t*). Once a significant amount of evidence is accumulated, further evidence will barely increase certainty about the true rewards. Thus, it becomes more preferable to decide quickly rather than to withhold choice for an insignificant increase in choice accuracy (even for similar reward estimates, 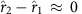, and residual uncertainty about which option yields the higher reward). The narrowing boundary separation ensures such rapid decisions (Fig. 4c for large *t*).

We can further simplify the optimal decision procedure by implementing the computation of the expected option reward difference by a diffusion model. As long as 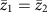, such an implementation remains statistically optimal, as the diffusing particle, *x*(*t*)≡*x*_1_(*t*)−*x*_2_(*t*), (recall that 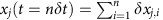) and elapsed time *t* form a set of sufficient statistics of the posterior *r*_1_(*t*)−*r*_2_(*t*)|*δ**x***(0:*t*) over this difference (see Methods section). Furthermore, *x*_*j*_(*t*) can be interpreted as the sample path of a particle that diffuses with variance *σ*^2^ and drifts with rate *z*_*j*_. For this reason, *x*(*t*) diffuses with variance 2*σ*^2^ and drifts with rate *z*_1_−*z*_2_, thus forming the particle in a diffusion model that performs statistically optimal inference. The same mapping between expected reward difference and diffusing particle allows us to map the optimal boundary on reward into boundaries on *x*(*t*) (Fig. 4c,d). Therefore, models as simple as diffusion models can implement optimal value-based decision-making.

### Moving from single choices to a sequence thereof

So far we have focused on single choices in which the decision maker trades off the expected reward received for this choice with the cost associated with accumulating evidence about the true option rewards. This setup assumes a single choice and, besides the accumulation cost, infinite time to perform it. In realistic scenarios, however, such choices are usually embedded within a sequence of similar choices. Here, we consider how such embedding influences the form of the optimal policy.

*Maximizing the reward rate across choices*. We assume that each choice within the sequence follow the previous single-choice setup. That is, after onset of the choice options, the decision maker pays a cost *c* per second for accumulating evidence about the true option rewards. At choice, she receives the true reward associated with the chosen option. The choice is followed by a (possibly stochastic) waiting time of *t*_*w*_ seconds on average, after which two new choice options appear and new evidence is accumulated. The true reward associated with either option is before choice option onset drawn according to the previously described Gaussian prior (Fig. 2a), such that these rewards remain constant within individual choices, but vary across consecutive choices. Rather than maximizing the total expected reward for each individual choice, we assume that the aim is to maximize the total expected reward within a fixed time period, independent of how many choices are performed within this period. To avoid boundary effects, we assume the period duration to be close-to-infinite, such that maximizing the total expected reward within this period becomes equivalent to maximizing the reward rate *ρ*, given by


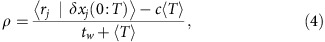


where the expectation is, as for equation (2), across choices *j* and evidence accumulation times *T*, given the flow of evidence. Here, it is critical that we fix the time period while leaving open the number of choices that can be performed. If we instead were to fix the number of choices while leaving open the time to make them, it again becomes optimal to maximize the total expected reward for each of these choices separately, such that the optimal policy for each such choice is the same as that for single, isolated choices.

Infinite choice sequences make using the standard value function difficult. This value function returns the total expected reward for all current and future choices when starting from the current state. For an infinite number of such future choices, the value function might thus become infinite. One way to avoid this is to use instead the ‘average-adjusted value' function, which—in addition to an accumulation cost—penalizes the passage of some time duration *δt* by −*ρδt*, where *ρ* is the reward rate. This reward rate is by equation (4) the total reward received (including accumulation costs) per second, averaged over the whole choice sequence. Penalizing the value function by this reward rate makes explicit the implicit loss of rewards due to potential future choices that the decision maker misses out on when accumulating too much evidence for the current choice. This penalization allows us to treat all choices in the sequence as if they were the same, unique choice. A further consequence of this penalization is that the value function for accumulating more evidence for some duration *δt* undergoes a more significant change, as accumulating this evidence now comes at a cost −(*c*+*ρ*)*δt* instead of the previous −*cδt* (see Methods section for the associated Bellman equation). For positive reward rates, *ρ*>0, this cost augmentation implies more costly evidence accumulation such that it becomes advantageous to accumulate less evidence than for single, isolated choices. This change is implemented by decision boundaries that collapse more rapidly (shown formally in Supplementary Note 1, see also Supplementary Fig. 1). Thus, collapsing decision boundaries implement the optimal policy for both single choices and sequences of choices, with the only difference that these boundaries collapse more rapidly for the latter. The duration of inter-choice waiting *t*_*w*_ modulates this difference, as with *t*_*w*_→∞, the reward rate described by equation (4) reduces to the expected reward for single, isolated choices, equation (2). Therefore the policy for single trials is a special case of that for maximizing the reward rate in which the waiting time between consecutive choices becomes close-to-infinite.

**Figure 5:**
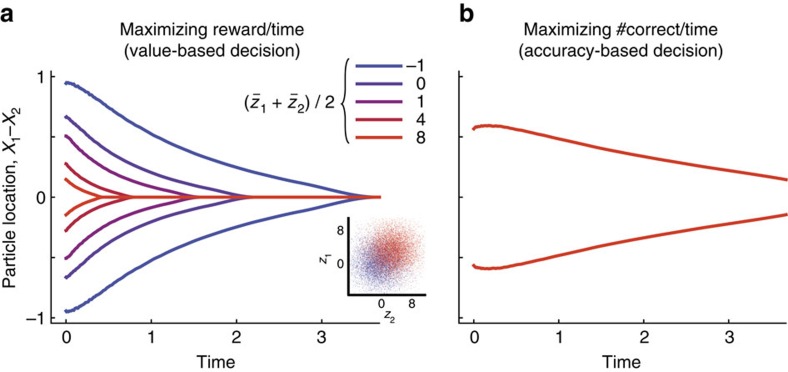
Effects of prior and task demands on the speed of boundary collapse. (**a**) The decision boundaries for value-based decisions that maximizes the reward per unit time. The average *a priori* reward 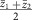 is varied from −1 to 8, while keeping 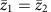. The inset shows two examples of prior distributions (blue and red: mean reward 0 and 4, respectively). The figure illustrates that the optimal decision boundaries depend on the prior knowledge about the average reward across trials. (**b**) The decision boundaries that maximizes the number of correct response per unit time for accuracy-based decisions. (The ‘correct rate' in value-based decisions can be defined, for example, as the probability of choosing the more preferable option.) These boundaries do not vary with 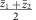 are all plotted on top of each other. Here, the decision boundaries were derived with the same dynamic-programming procedure as for the value-based case, except for that the rewards were assumed to be binary, and only one if the decision maker correctly identified the option with the larger ‘reward' cue *z*_*j*_ (see Methods section). In contrast to the reward-rate maximization strategy for value-based decisions (**a**), the decision strategy maximizing the correct rate is invariant to the absolute values of mean reward/evidence strength, thus demonstrating a qualitative difference between value-based and perceptual decision-making in terms of the optimal strategy. In addition, the optimal boundaries in the value-based case approach each other more rapidly over time than for perceptual decisions. The faster boundary collapse for value-based decisions is consistent across a broad range of mean absolute rewards, showing that the distinction in boundary dynamics is not just due to the difference in expected reward rates, but reflecting a qualitative difference between the geometries of value functions in these two tasks.

*Dependency of the policy on the prior distribution of reward*. As shown above, optimal value-based decisions are achieved by accumulating only the difference of reward estimates, as implementable by DDMs. However, this does not mean that the absolute reward magnitudes have no effect on the decision strategy; they affect the decision boundary shape. Figure 5a shows how the optimal decision boundaries depend on the mean of the *a priori* belief about the true rewards across trials. When both options are likely to be highly rewarding on average, the boundaries should collapse more rapidly to perform more choices within the same amount of time. In the light of a guaranteed high reward, this faster collapse promotes saving time and effort of evidence accumulation. The boundary shape does not change for trial-by-trial variations in true rewards (which are *a priori* unknown) for the same prior, but only when the prior itself changes. This sensitivity to the prior and associated average rewards also differentiates reward rate-maximizing value-based decision-making from decisions that aim at maximizing the reward for single, isolated choices (Supplementary Note 1), and from classic paradigms of perceptual decision-making (Fig. 5b, see also Discussion section). To summarize, for value-based decisions that maximize the reward rate, the *a priori* belief about average-reward magnitudes affect the strategy (and, as a consequence, the average reaction time) by modulating the speed of collapse of the decision boundaries, even if choices within individual decisions are only guided by the relative reward estimates between options.

### The limits of diffusion models for value-based decisions

**Figure 6:**
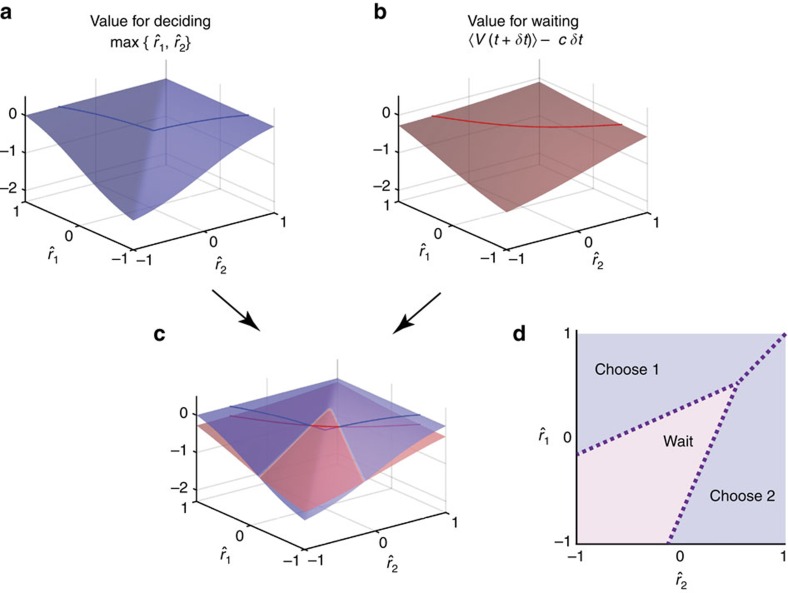
In some scenarios the optimal policy becomes even more complex than two parallel boundaries in the space of expected reward estimates. This property might, for example, break down if the utility that the decision maker receives from her choices is not the reward itself but instead a non-linear function of this reward. If this utility grows sub-linearly in the reward, as is frequently assumed, the decision boundaries approach each other with increasing expected reward, as higher rewards yield comparably less utility. In such circumstances, optimal choices require tracking of both expected reward estimates, 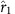 and 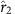, independently rather than only their difference. To demonstrate this, here we assumed a saturating utility function, *Utility*=*u*(*r*), which saturates at *r*→∞ and *r*→−∞. This could be the case, for example, if rewards vary over a large range over which the subjectively perceived utility follows a non-linear saturating function of this reward. (In this figure, *u* is modelled with a tangent hyperbolic function, but the exact details of the functional form do not qualitatively change the results). The logic of the different panels follows that of Fig. 2. (**a**) The value function surface for choosing either of two options. (**b**) The value surfaces for postponing decision to accumulate more evidence for a period of *δt*. (**c**) The two value surfaces superimposed. (**d**) The decision boundary and choice represented in the two-dimensional space of 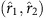. Note that the distance between decision boundaries is narrower in the regime where estimated rewards are high on average, resembling ‘RMs'^11^,^12^, which are more sensitive to absolute reward magnitudes than DDMs.

For all scenarios we have considered so far, diffusion models can implement the optimal decision-making policy. Here, we discuss that this is still the case for some, but not all generalizations of the task. For some tasks, the optimal policy won't even be representable by parallel boundaries in the 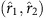-space of expected reward estimates. This is, for example, the case when the prior/likelihood distributions of reward/evidence are correlated in a particular way (see Methods section and Supplementary Note 1), or when the utility function is non-linear (see Fig. 6 for an example).

Thus, diffusion models only seem to implement the optimal decision strategy under very constrained circumstances. However, even beyond these circumstances, diffusion models might not be too far off from achieving close-to-optimal performance, but their loss of reward remains to be evaluated in general circumstances. Laboratory experiments could satisfy conditions for diffusion models to be close-to-optimal even in the presence of a non-linear utility function. Such experiments often use moderate rewards (for example, moderately valued food items, rather than extreme payoffs) in which case a potentially non-linear utility would be well-approximated by a linear function within the tested range of rewards.

## Discussion

We have theoretically derived the optimal behaviour for value-based decision-making with noisy evidence about rewards. Our analysis revealed that the optimal strategy in a natural problem setup (where values are linear in rewards) reduces to a DDM with time-varying boundaries. This result provides a theoretical basis for why human decision makers seem to feature behaviour in such tasks that, just as in accuracy-based (conventional perceptual) decisions, is well captured by DDMs—despite the profound qualitative difference in task structures (for example, a two-dimensional value functions for value-based tasks, but not for accuracy-based ones). Furthermore, we found that the optimal strategy does not always reduce to DDMs if we assume non-linear relationships between value and reward (Fig. 6), predicting that human behaviour may deviates from DDMs in specific experimental conditions (perceived utility following a non-linear saturating function of this reward; Fig. 6d); interestingly, such decision boundary structure might be better approximated by ‘correlated RMs'^11^,^12^.

Simultaneous to our work, another theoretical study by Fudenberg *et al*. (unpublished work^22^) has recently focused on optimal evidence accumulation and decision-making for value-based decisions. This study provides a more in-depth mathematical characterization of the optimal policy implemented by diffusion model with collapsing boundaries. Their analysis, however, is restricted to single, isolated choices, and—unlike us—does not consider policy changes for reward rate maximization, nor non-linear utility functions that invalidate the use of diffusion models.

Whether human and animal use collapsing decision boundaries is a topic of debate in the recent accuracy-based^23^ and value-based^9^ decision-making studies. Interestingly, a recent meta-analysis study reports that whether subject uses collapsing boundaries varies strongly across tasks and individuals^23^. Our theory suggests that the optimal boundary dynamics is sensitive to task demands (for example, reward-rate maximization or correct-rate maximization) as well as the absolute mean reward magnitude (in contrast to perceptual decision-making; see Supplementary Note 2). Thus, subjects might switch their decision strategies depending on those experimental factors, emphasizing the need to carefully control these factors in further studies.

Still, in both daily lives and laboratory experiments, humans can sometimes take a long time to decide between two valuable options, which might reflect suboptimal behaviour or an insufficiently fast collapse of the bound. For instance, a recent empirical study by Oud *et al*.^24^ reports slower-than-optimal value-based and perceptual choices of human decision makers in a reward rate maximization setting. These slow choices might arise, however, from incompletely or incorrectly learned priors (Supplementary Note 3), and warrant further investigation. Another slowing factor is insufficient time pressure induced by, for example, fixing the number of choices instead of the total duration of the experiment. In this case, the slow reaction times may not reflect a suboptimal strategy. For example, Milosavljevic *et al*.^9^ have found that subjects can take a surprisingly long time to decide between two high-valued items but, in this experiment, subjects had to perform a fixed number of choices without any time constraint. Their reward at the end of the experiment was determined by drawing one item among all the items selected by the subject^9^. With such a task design, there is no explicit incentive for making fast choices and, therefore, the optimal strategy does allow for long reaction times. All of the above cases highlight that the seeming irrationality of slow choices between two high-valued options might in fact reflect a completely rational strategy under contrived laboratory settings. Thus, by revealing the optimal policy for value-based decisions, the present theory provides a critical step in studying the factors that determine our decisions about values.

What do collapsing boundaries in diffusion models tell us about the neural mechanisms involved in such decisions? Previous studies concerning perceptual decisions have linked such boundary collapse to a neural ‘urgency signal' that collectively drives neural activity towards a constant threshold^7^,^25^. However, note that in such a setup even a constant (that is, non-collapsing) diffusion model bound realizes a collapsing bound in the decision maker's posterior belief^7^. Analogously, a constant diffusion model bound in our setup realizes a collapsing bound on the value estimate difference. Furthermore, how accumulated evidence is exactly coded in the activity of individual neurons or neural populations remains unclear (for example, compare refs 6, 26), and even less is known about value encoding. For these reasons we promote diffusion models for behavioural predictions, but for now refrain from directly predicting neural activity and associated mechanisms. Nonetheless, our theory postulates what kind of information ought to be encoded in neural populations, and as such can guide further empirical research in neural value coding.

## Methods

### Structure of evidence and evidence accumulation

Here, we assume a slightly more general version of the task than the one we discuss throughout most of the main text, with a correlated prior and a correlated likelihood. Further below we describe how this version relates to the one in the main text. In particular, we assume the prior over true rewards, given by vector 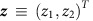, to be a bivariate Gaussian, 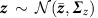, with mean 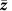 and covariance ***Σ***_*z*_. In each small time step *i* of duration *δt*, the decision maker observes some momentary evidence 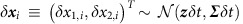 that informs her about these true rewards. After accumulating evidence for some time *t*=*nδt*, her posterior belief about the true rewards is found by Bayes' rule, 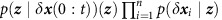, and results in


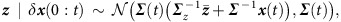


where we have defined 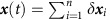 as the sum of all momentary evidence up to time *t*, and 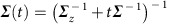 as the posterior covariance (hereafter, when ***Σ***(*t*) is a function of time it denote the posterior covariance, rather than the covariance of evidence, ***Σ***). For the case that experienced reward ***r***≡(*r*_1_, *r*_2_)^*T*^ equals true reward ***z***, that is ***r***=***z***, the mean estimated option reward 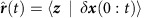 is the mean of the above posterior.

### Expected future reward estimates

Finding the optimal policy by solving Bellman's equation requires computing the distribution of expected future rewards 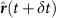 given the current expected rewards 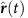. Assuming a small *δt* such that the probability of an eventual boundary crossing becomes negligible, we can find this distribution by the marginalization


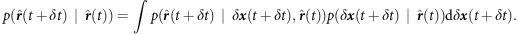


As 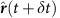 is the mean of the posterior of ***z*** after having accumulated evidence up to time *t*+*δt*, it is given by


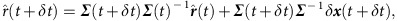


where we have used ***x***(*t*+*δt*)=***x***(*t*)+*δ**x***(*t*+*δt*) and 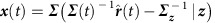, following from the definition of 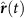. Furthermore, by the generative model for the momentary evidence we have 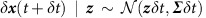, and our current posterior is 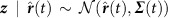, which, together, gives 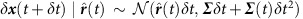. With these components, the marginalization results in


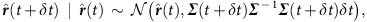


where we have only kept terms of order *δt* or lower. An extended version of this derivation is given in Supplementary Note 1.

### More specific task setups

Here, we consider two more specific task setups. In the first one, the prior covariance is proportional to the likelihood covariance, that is ***Σ***_*z*_=*α**Σ***. This causes the posterior ***z*** to be given by


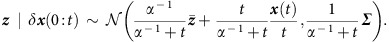


In this case, the posterior mean becomes independent of the covariance, and is a weighted mixture of prior and accumulated evidence. The distribution over expected future reward estimates becomes 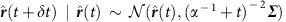. In terms of choosing among lunch menus, a positively correlated prior could correspond to differently skilled cooks working on different days, such that the true rewards associated with the different options fluctuate jointly. A correlated likelihood might correspond to fresh produce in one menu option predicting the same in the other menu option. If the likelihood covariance is proportional to that of the prior, diffusion models still implement the optimal choice policy.

In the second more specific setup we assume both prior and likelihood to be uncorrelated, with covariance matrices given by 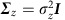 and ***Σ***=*σ*^2^***I***. This is the setup discussed throughout most of the work, and results in an equally uncorrelated posterior ***z***, that is for option *j* given by equation (1). The distribution over expected future reward estimates is also uncorrelated, and for option *j* is given by 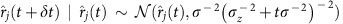.

A more general scenario than the ones we have discussed so far is that both the decision-maker's *a priori* belief about the true rewards, as well as the likelihood of the momentary evidence about these rewards are correlated, but the prior covariance is not proportional to the likelihood covariance. Once prior covariance and likelihood covariance are not proportional to each other anymore, diffusion models fail to implement the optimal policy. Even then, the optimal policy in the 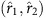-space of expected reward estimates is still given by two boundaries parallel to the identity line, such that we can again only bound the difference between these estimates. However, these are bounds on expected reward estimate differences, and not on a diffusing particle. Mapping the estimates into a single diffusing particle requires combining them linearly with combination weights that change over time, which is incompatible with the standard diffusion model architecture (although it can be implemented by an extended diffusion model as shown in (ref. 27). Thus, parallel decision boundaries on expected reward estimates do not automatically imply that diffusion models can implement optimal decisions.

### Evidence accumulation and decisions with diffusion models

For the class of tasks in which the decision boundaries in 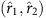 are parallel to the diagonal, the optimal policy can be represented by two boundaries, *ξ*_1_(*t*) and *ξ*_2_(*t*), on the expected reward difference 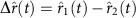, such that evidence is accumulated as long as 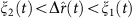, and option 1 (option 2) is chosen as soon as 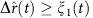 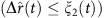. To implement this policy with diffusion models, we need to find a possibly time-dependent function 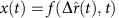 that maps the expected reward difference 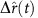 into a drifting/diffusing particle d*x*(*t*)=*μ*d*t*+*σ*_*x*_d*W*_*t*_, that drifts with drift *μ* and diffuses with variance 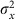, and where d*W*_*t*_ is a Wiener process. Such a mapping allows us to find the boundaries 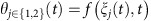, that implement the same policy by bounding particle *x*(*t*).

For the general case of a correlated prior and correlated likelihood, as discussed further above, we have 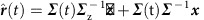, where ***x***(*t*) drifts and diffuses according to 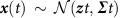. Using 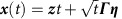, where 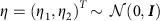, and a ***Γ*** that satisfies ***ΓΓ***^*T*^=***Σ***, we find


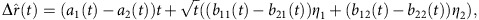


with *a*_*j*_(*t*) denoting the elements of vector 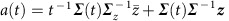 and *b*_*ij*_(*t*) being the elements of matrix 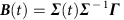. The above describes a diffusion process with drift and diffusion that vary over time in different ways. Therefore, we cannot find a function *f*(·, *t*) that maps 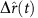 into a diffusing particle with constant drift and diffusion. As a result, we cannot use diffusion models for optimal decision-making in this case.

One reason for this incompatibility is that the posterior covariance changes from prior covariance, 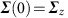, to likelihood covariance, 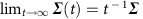, over time and influences the relation between drift and diffusion. If we set the prior covariance proportional to the likelihood covariance, that is 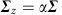, then we can find a mapping to diffusion models. Using the mean of the posterior ***z*** from the previous section, we find that 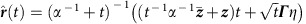, which results in the expected reward difference


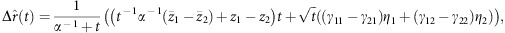


where *γ*_*ij*_ are the elements of ***Γ***. Now, as long as 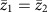 (a priori, both options have the same true reward), we can use the mapping 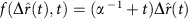 to map the boundaries in the diffusion model space, which features a particle that drifts with drift *μ*=*z*_1_−*z*_2_ and diffuses with variance 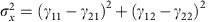.

The setup that is discussed throughout the main text becomes even simpler, with a diagonal prior covariance, 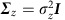, and a diagonal likelihood covariance 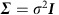. Using the mean of the posterior in equation (1), and again assuming 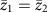, a similar argument as before shows that the mapping 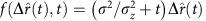 allows us to implement optimal decision-making with a diffusion model with drift *μ*=*z*_1_−*z*_2_ and diffusion variance 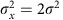.

### Bellman's equation for single isolated trials

The rationale behind optimal decision-making in single, isolated trials is explained in the main text and is here repeated only briefly. At each point in time *t* after onset of the choice options, the decision maker performs the action that promises the largest sum of expected rewards from that point onwards (including the cost for accumulating evidence). Given that at this time the decision maker holds a posterior belief over values with sufficient statistics 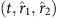, the sum of expected rewards is denoted by the value function 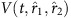. The available actions are to either choose option one or two, or to accumulate more evidence and decide later. Deciding immediately, the decision maker would choose the option that is expected to yield higher reward, such that the value associated with deciding is 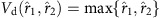. Accumulating evidence for another time period *δt* comes at cost *cδt* but is expected to yield reward 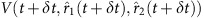. Here, the expectation is over how the sufficient statistics are expected to evolve when accumulating more evidence, and is given by the bivariate Gaussian 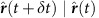 that we have derived further above. Thus, the value for waiting is 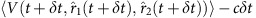. At any time *t*, the decision maker chooses the action associated with the higher value, which leads to Bellman's equation, as given by equation (3) in the main text. This equation on one hand defines the value function, and on the other hand determines the optimal policy: as long as the value for waiting dominates, the decision maker ought to accumulate more evidence. Once the value for deciding becomes larger, it is best to choose the option that is expected to yield the higher reward.

### Bellman's equation for reward rate maximization

In order to find Bellman's equation and the associated optimal policy that maximizes the reward rate, we borrow concepts from average-reward DP (refs 7, 28). We do so to avoid that the value function associated with the first trial becomes infinite if this trial is followed by an infinite number of trials that, in total, promise infinite reward. Average-reward DP penalizes the passage of some time *δt* by cost *ρδt*, where *ρ* is the reward rate, equation (4), which equals the average expected reward per unit time. With this additional cost, and the value function turns into the ‘average-adjusted value' function 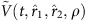, which is the same for each trial in the sequence, and is defined as follows. Immediate decisions are expected to be rewarded by 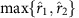, followed by some waiting time *t*_*w*_ that comes at cost *ρt*_*w*_. After this waiting time, the decision maker holds belief 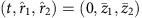 (recall that 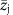 denotes the prior mean for option *j*) at the onset of the next trial, and therefore expects reward 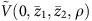 in this trial. Thus, the value for decision immediately is given by 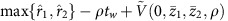. The value for accumulating more evidence is the same as for single, isolated trials (see previous section), only that the cost increases from *cδt* to (*c*+*ρ*)*δt*. Bellman's equation is again given by taking the maximum over all values. In contrast to single, isolated trials, the policy arising from Bellman's equation is invariant to global shifts in the value function. That is, we can add some constant *C* to the average-adjusted value associated with all sufficient statistics, such that 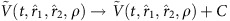, and would recover the same policy^28^. As a result, we can arbitrarily fix the average-adjusted value for one such statistic, and all other values follow accordingly. For convenience, we choose 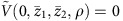, which results in Bellman's Equation


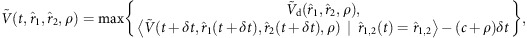


where 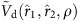 is given by 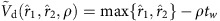. This also gives us a recipe to find 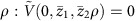 will only hold for the correct *ρ*, such that we can compute 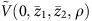 for some arbitrary *ρ*, and then adjust *ρ* until 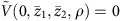 holds. This is guaranteed to provide the desired solution, as 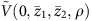 is strictly decreasing in *ρ* as long as *t*_*w*_>0 (rather than *t*_*w*_=0; see Supplementary Note 1).

### Bellman's equation for maximizing the correct rate

We now move to assuming that, all that matters to the decision maker is to identify the higher rewarded option, irrespective of the associated true reward. To do so, we abolish the identity between true and experienced reward, *z* and *r*, and instead assume that an experienced reward of *r*=*R*_corr_ is associated with choosing option *j* if *z*_*j*_>*z*_*i*_, *i*≠*j*, and a reward of *r*=*r*_incorr_ with the alternative choice. This captures the case of maximizing the correct rate for value-based decisions, and also relates closely to simpler perceptual decisions in which the decision maker only gets rewarded for correct choices (for example, ref. 29), as long as the momentary evidence is well-approximated by a Gaussian. Evidence accumulation in this setup remains unchanged from before, as the posterior 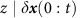 contains all information required to compute the expected experienced reward. This posterior is fully specified by the sufficient statistics 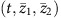, where we have defined 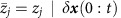.

The value function for single, isolated trials changes in two ways. First, it is now defined over 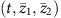 instead of 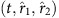 (previously we had 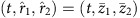, which does not hold anymore). Second, the value for deciding changes as follows. When choosing option one, the decision maker receives reward *R*_corr_ with probability 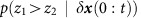 and reward *R*_incorr_ with probability 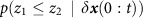. Thus, the expected reward associated with this choice is 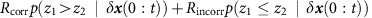. The expected reward for option two is found analogously, and results in the value for deciding


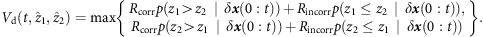


As the posterior 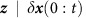 is Gaussian in all task setups we have considered, the probabilities in the above expression are cumulative Gaussian functions that are functions of the sufficient statistics 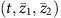. Besides these two changes, the value function and associated Bellman Equation remain unchanged (see Supplementary Note 1 for explicit expressions).

Moving from single, isolated trials to maximizing the correct rate over sequences of trials requires the same changes as when moving from single trials to maximizing the reward rate. In particular, the value function turns into the average-adjusted value function that penalizes the passage of some time *δt* by *ρδt*, where *ρ* is now the correct rate rather than the reward rate. The correct rate is still the average experienced reward (minus accumulation cost) per unit time, but—due to the changed definition of experienced reward—does not anymore relate to the true reward, but only if the option associated with the larger associated true reward was correctly identified. This causes the value for deciding to be additionally penalized by *ρt*_*w*_. The value for waiting some more time *δt* to accumulate more evidence incurs an additional cost *ρδt*, but remains unchanged otherwise. The average-adjusted value function is again invariant under addition of a constant, such we choose 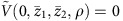. This fully specifies the value function and associated Bellman equation, which is provided in Supplementary Note 1.

### Linearity of value function for waiting

Here, we show that value function for waiting increases linearly in line parallel to the diagonal within the 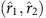-space, which is required to show that the optimal decision boundaries are parallel to the diagonal. We will do so by a backwards induction argument in time. The base case for the induction argument relies on the shape of the value function for large times, *t*→∞. For such times, the decision maker incurs a large cost for accumulating evidence up until that time, and also expects to gain little further insight into the true rewards when accumulating more evidence. As a consequence, at such times it will always be better to decide immediately rather than to accumulate more evidence. Therefore, the value function will be given by the value for deciding, 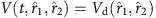, which, as discussed in the previous paragraph, is linearly increasing in lines parallel to the diagonal.

The inductive step will show that, if the value function at time *t*+*δt* is linearly increasing in lines parallel to the diagonal, then so it the value of waiting at time *t*, and, as a consequence, also the value function at time *t*. The value of waiting at time *t* is given by 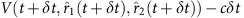, where the expectation is over future expected rewards 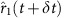 and 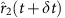, and reflects the uncertainty about how the reward estimate evolves over time. For our case, the distribution describing this uncertainty is a bivariate Gaussian (as described in the previous sections), centred on the current expected rewards, 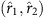, and with a covariance that only depends on *t*. Its shift-invariant shape causes the expectation 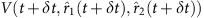 to be a smoothed version of 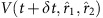 that, as 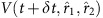, linearly increase in lines parallel to the diagonal. The value of waiting is this expectation shifted by the constant momentary cost −*cδt*, and therefore also has this property (Fig. 3b). This establishes that, if the value function at time *t*+*δt* is linearly increasing in lines parallel to the diagonal, then so is the value of waiting at time *t*. The value function at time *t* is the maximum over the value for deciding and that for waiting. As both increase linearly in lines parallel to the diagonal, so does this value function, 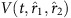 (Fig. 3c,e). This completes the inductive step.

The induction argument shows that both value for deciding as well as that for waiting increases linearly with slope one in lines parallel to the diagonal for all *t*. This immediately means that, if they intersect at some point 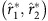, then they will intersect at the whole line 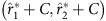 that is parallel to the diagonal (Fig. 3c). As a consequence, the decision boundaries, which lie on the intersection between these two values, are parallel to this diagonal for all times *t*. See Supplementary Note 1 for the proof of the same property with an argument that does not rely on induction.

### Finding the optimal policy numerically

To find the optimal policy for the above cases numerically, we computed the value function by backward induction^30^, using Bellman's equation. Bellman's equation expresses the value function at time *t* as a function of the value function at time *t*+*δt*. Therefore, if we know the value function at some time *T*, we can compute it at time *T*−*δt*, then *T*−2*δt*, and so on, until time *t*=0. We usually chose some large T, significantly beyond the time horizon of interest, at which we set 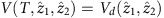, independent of the value at any *t*>*T*. For any time *t*≤*T*, we represented the value function over the remaining two parameters 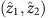 (or 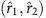 in the value-based task) numerically over an equally space two-dimensional grid. This grid allowed us to compute the integral that represents the expectation over the future value numerically by the two-dimensional convolution between future value Vt+δt,1t+δt,z2t+δt and transition probability distribution 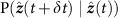. For any such time, the optimal decision boundaries were found on this grid by the intersection of the value for deciding and that for waiting. We handled boundary effects in space and time by significantly extending the grid beyond the area of interest and cropping the value function after fully computing it over the extended range.

In the reward rate and correct rate case, computing the value function requires knowledge of the corresponding rate *ρ*. This *ρ* was unknown, but could be found by the condition 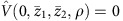. 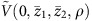 is strictly decreasing in *ρ* (Supplementary Note 1), such that we could initially assume an arbitrary *ρ* for which we computed 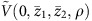. The correct *ρ* was then found by iterating the computation of 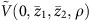 within a root finding procedure until 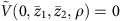.

The following parameters were used to generate the figures. We set the prior mean as 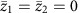, except for Fig. 5 where we varied 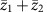 while fixing 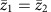. The prior variance was 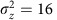, and observation noise 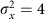, for both options. We used a grid spanning 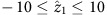 and 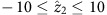, in steps of 0.4 in both dimensions. The maximum time to consider was set to *T*=5 s, with time-steps of size *δt*=0.005 s for backward induction. To focus on the effect of reward rate, we assumed no explicit cost of evidence accumulation, *c*=0 and a waiting time *t*_*w*_ set to 0.5 s.

### Data availability

The authors declare that the data supporting the findings of this study are available within the article and its Supplementary Information File.

## Additional information

**How to cite this article:** Tajima, S. *et al*. Optimal policy for value-based decision-making. *Nat. Commun.* 7:12400 doi: 10.1038/ncomms12400 (2016).

## Supplementary Material

Supplementary Information Supplementary Figure 1, Supplementary Notes 1-3 and Supplementary References

## Figures and Tables

**Figure 1:**
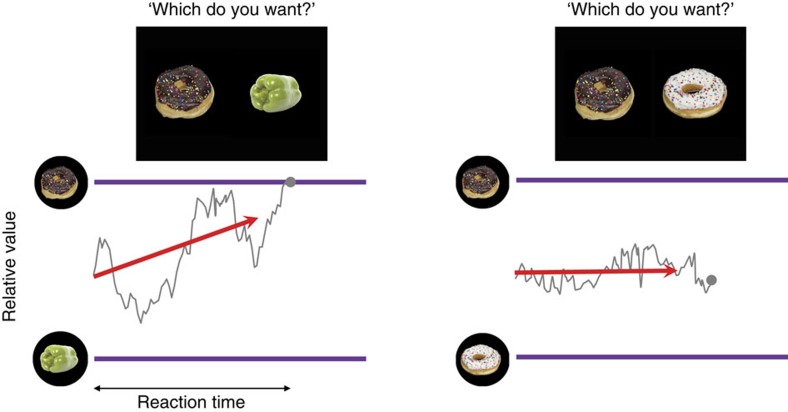
DDMs for value-based decisions. The purple lines represent the decision boundaries. The red arrows indicate the mean drift rate. The grey fluctuating traces illustrate sample trajectories of a particle that drifts in the space of relative value between two given options. (left) If an option is preferred (that is, yields higher reward) than the other, the mean drift is biased toward the boundary of preferred option, making the particle to hit the decision boundary within a relatively short time. (right) However, if the given options are equally good, DDM assumes a mean drift without any bias, requiring much longer time for the particle to hit either decision boundary—even if both options are highly rewarding.
