# Supplementary material for: Optimal policy for value-based decision-making
Source: Nat Commun. 2016 Aug 18;7:12400. doi: 10.1038/ncomms12400 (PMC4992126; doi:10.1038/ncomms12400)
Supplement: Supplementary Information — Supplementary Figure 1, Supplementary Notes 1-3 and Supplementary References [file ncomms12400-s1.pdf]

Supplementary Figure

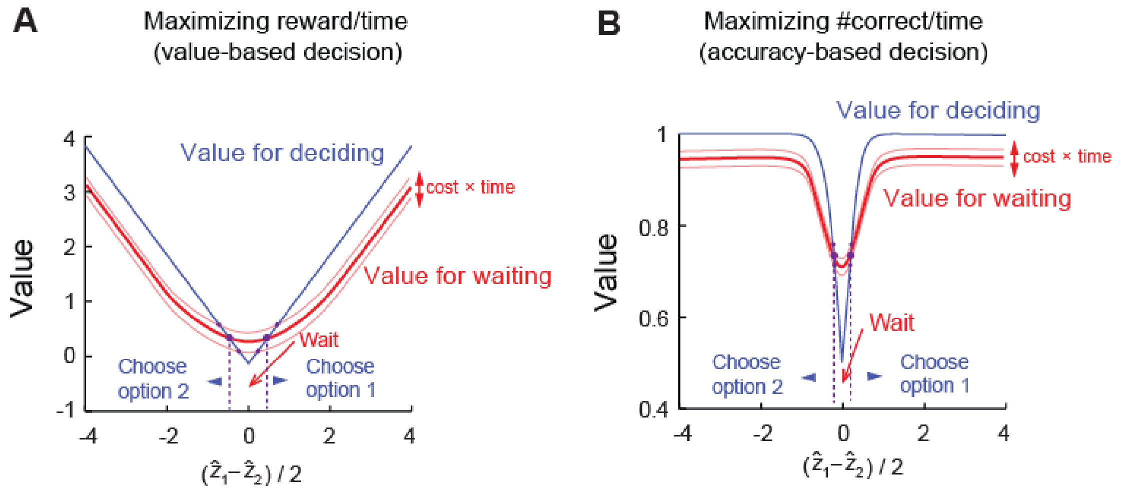

Supplementary Figure 1

Geometric explanation of the reason why the value-based decisions lead to more rapid boundary collapse than the accuracy-based decisions. The figures depict the values for "deciding" (blue) and for "waiting" (red) in value-based and in accuracy-based decisions. Sections at a $\hat{z}_1 + \hat{z}_2 =$ const., as in **Fig. 3D**.

(A) In the value-based decisions, the value functions for deciding and for waiting are both effectively linear and parallel to each other in the most of regime. The value for deciding does not depend on time, as it is max{ $\hat{z}_1, \hat{z}_2$} by definition. The value for waiting is a smoothed and shifted version of it, reflecting the uncertainty about future evidence and cost of evidence accumulation. When the value for waiting changes (as illustrated by the thin red curves in the figure), the intersections of two value functions moves along the value of deciding. Because the two value functions (blue and red) are nearly parallel, loci of intersections between them are sensitive to the uncertainty and cost of waiting (multiplied by time), causing the fast dynamics of the decision boundaries.

(B) In the accuracy-based decisions, in contrast, the expected reward reflects the probability of making a correct choice, which is computed as the maximum of two cumulative Gaussian functions. As a result, the value function for deciding has concave parts and a sharp valley between them (at $\hat{z}_1 = \hat{z}_2$). The value for waiting is a smoothed and shifted version of it, as in the value-based case (A), but now more likely to have intersections with the value function for deciding, due to the sharp valley between the two concave parts. Note that, at the positive and negative extremes of $\hat{z}_1 - \hat{z}_2$, the value for waiting is always under the one for deciding due to the cost of evidence accumulation. Thus, if the two value functions have intersections, the loci of those intersections are restricted around the valley. This leads to stability of the decision, resulting in slow dynamics of decision boundaries. Note that the more rapid collapse of the decision boundary in the value-based case does not necessarily imply *earlier* choices; when the boundaries are initially further apart (as for smaller average-reward conditions, blue lines in **Fig. 5A**), decisions can be slower even though the boundaries approach each other more rapidly.

Supplementary Note 1

Evidence accumulation

Task elements

Here we focus on the most general version of the task. All task versions discussed in the main text are specialized versions of the task described here, and therefore share its properties. This section is similar to the first section in **Methods** of the main text, and is replicated here for the sake of completeness.

Let $\boldsymbol{z} \equiv (z_1, z_2)^T$ denote the true reward associated with choice options 1 and 2. This true reward varies across choices/trials according to a bivariate Gaussian distribution $\boldsymbol{z} \sim \mathcal{N}(\bar{\boldsymbol{z}}, \boldsymbol{\Sigma}_z)$ with mean $\bar{\boldsymbol{z}} \equiv (\bar{z}_1, \bar{z}_2)^T$ and covariance $\boldsymbol{\Sigma}_z$. The decision maker knows this distribution, but never directly observes the true reward associated with either choice option. Instead, in every small time step i of duration δt, she observes some momentary evidence $\delta \boldsymbol{x}_i \equiv \left(\delta x_{1,i}, \delta x_{2,i}\right)^T \sim \mathcal{N}(\boldsymbol{z}\delta t, \boldsymbol{\Sigma}\delta t)$ that informs her about the true reward. After accumulating evidence for some time $t = n\delta t$, her posterior belief about the true reward is found by Bayes' rule, $p\left(\boldsymbol{z}|\delta \boldsymbol{x}(0{:}\,t)\right) \propto p(\boldsymbol{z}) \prod_{i=1}^{n} p(\delta \boldsymbol{x}_i|\boldsymbol{z})$, and results in

$$\boldsymbol{z}|\delta \boldsymbol{x}(0{:}\,t) \sim \mathcal{N}\left(\boldsymbol{\Sigma}(t)\left(\boldsymbol{\Sigma}_z^{-1}\bar{\boldsymbol{z}} + \boldsymbol{\Sigma}^{-1}\boldsymbol{x}(t)\right), \boldsymbol{\Sigma}(t)\right),$$

where we have defined $\boldsymbol{x}(t) = \sum_{i=1}^{n} \delta \boldsymbol{x}_i$ as the sum of all momentary evidence up to time t, and $\boldsymbol{\Sigma}(t) = (\boldsymbol{\Sigma}_z^{-1} + t\boldsymbol{\Sigma}^{-1})^{-1}$ as the posterior covariance. With $\delta t \to 0$, the process becomes continuous in time, such that $\boldsymbol{x}(t)$ becomes the integrated momentary evidence, but the above posterior still holds.

For most of the below we assume that reward $\boldsymbol{r}$ experienced by the decision maker upon choosing one of the two options equals the true reward, that is $\boldsymbol{r} = \boldsymbol{z}$. Therefore, the mean estimated option reward, $\hat{\boldsymbol{r}}(t) = \langle \boldsymbol{z}|\delta \boldsymbol{x}(0{:}\,t)\rangle$ is the mean of the above posterior.

The expected reward process

Finding the optimal policy involves predicting how the mean estimated option reward might evolve if we accumulate more evidence. In this section we derive the stochastic process that describes this evolution. This section is an extended version of the second section in **Methods** of the main text.

Assume that having accumulated evidence until time t, the current expected reward is given by $\hat{\boldsymbol{r}}(t)$. In the absence of observing more evidence, the decision maker's prediction of how this expected reward might change when (hypothetically) accumulating more evidence for some duration δt is given by the marginalization

$$p(\hat{\boldsymbol{r}}(t+\delta t)|\hat{\boldsymbol{r}}(t)) = \int p(\hat{\boldsymbol{r}}(t+\delta t)|\delta \boldsymbol{x}(t+\delta t), \hat{\boldsymbol{r}}(t)) p(\delta \boldsymbol{x}(t+\delta t)|\hat{\boldsymbol{r}}(t)) \mathrm{d}\delta \boldsymbol{x}(t+\delta t).$$

That is, given current knowledge about $\boldsymbol{z}$, the decision maker hypothesizes the value of the next momentary evidence $\delta \boldsymbol{x}(t+\delta t)$, and uses this value to predict her updated estimate $\hat{\boldsymbol{r}}(t+\delta t)$. This updated estimate forms by definition the mean of $\boldsymbol{z}|\delta \boldsymbol{x}(0{:}\,t + \delta t)$, and is therefore given by $\hat{\boldsymbol{r}}(t+\delta t) = \boldsymbol{\Sigma}(t+\delta t)(\boldsymbol{\Sigma}_z^{-1}\boldsymbol{z} + \boldsymbol{\Sigma}^{-1}\boldsymbol{x}(t+\delta t))$. Furthermore, we have $\boldsymbol{x}(t+\delta t) = \boldsymbol{x}(t) + \delta \boldsymbol{x}(t+\delta t)$ and $\boldsymbol{x}(t) = \boldsymbol{\Sigma}(\boldsymbol{\Sigma}(t)^{-1}\hat{\boldsymbol{r}}(t) - \boldsymbol{\Sigma}_z^{-1}\boldsymbol{z})$ (following from $\hat{\boldsymbol{r}}(t)$ being the mean of $\boldsymbol{z}|\delta \boldsymbol{x}(0{:}\,t)$), such that $\hat{\boldsymbol{r}}(t+\delta t)$ relates to $\delta \boldsymbol{x}(t+\delta t)$ by

$$\hat{\boldsymbol{r}}(t+\delta t) = \boldsymbol{\Sigma}(t+\delta t)\boldsymbol{\Sigma}(t)^{-1}\hat{\boldsymbol{r}}(t) + \boldsymbol{\Sigma}(t+\delta t)\boldsymbol{\Sigma}^{-1}\delta \boldsymbol{x}(t+\delta t).$$

This shows that $p(\hat{\boldsymbol{r}}(t+\delta t)|\delta\boldsymbol{x}(t+\delta t),\hat{\boldsymbol{r}}(t))$, which is the first density in the above marginalization, is a delta-function.

The second term in the marginalization follows from the generative model for the momentary evidence, $\delta\boldsymbol{x}(t+\delta t)|\boldsymbol{z} \sim \mathcal{N}(\boldsymbol{z}\delta t, \boldsymbol{\Sigma}\delta t)$. Note that $\boldsymbol{z}$ itself is unknown, but the decision maker's current belief about $\boldsymbol{z}$ is $\boldsymbol{z}|\hat{\boldsymbol{r}}(t) \sim \mathcal{N}(\hat{\boldsymbol{r}}(t), \boldsymbol{\Sigma}(t))$. Therefore, we find $\delta\boldsymbol{x}(t+\delta t)|\hat{\boldsymbol{r}}(t)$ by marginalizing over $\boldsymbol{z}$, which results in $\delta\boldsymbol{x}(t+\delta t)|\hat{\boldsymbol{r}}(t) \sim \mathcal{N}(\hat{\boldsymbol{r}}(t)\delta t, \boldsymbol{\Sigma}\delta t + \boldsymbol{\Sigma}(t)\delta t^2)$.

With these two term in place, we can perform the marginalizing, which results in

$$\hat{\boldsymbol{r}}(t+\delta t)|\hat{\boldsymbol{r}}(t) \sim \mathcal{N}(\hat{\boldsymbol{r}}(t), \boldsymbol{\Sigma}(t+\delta t)\boldsymbol{\Sigma}^{-1}\boldsymbol{\Sigma}(t+\delta t)\delta t),$$

where we have used $\boldsymbol{\Sigma}(t)^{-1} + \delta t\boldsymbol{\Sigma}^{-1} = \boldsymbol{\Sigma}(t+\delta t)^{-1}$ to find the mean, and in the variance we have only kept terms of order δt or lower. Therefore, the change in this estimate has density

$$\hat{\boldsymbol{r}}(t+\delta t) - \hat{\boldsymbol{r}}(t)|\hat{\boldsymbol{r}}(t) \sim \mathcal{N}(\boldsymbol{0}, \boldsymbol{\Sigma}(t+\delta t)\boldsymbol{\Sigma}^{-1}\boldsymbol{\Sigma}(t+\delta t)\delta t),$$

This is true for any $t \geq 0$, such that with $\delta t \to 0$, $\hat{\boldsymbol{r}}(t)$ evolves according to the martingale

$$d\hat{\boldsymbol{r}}(t) = \boldsymbol{\Gamma}(t)d\boldsymbol{B}_t,$$

where $d\boldsymbol{B}_t$ is a two-dimensional Wiener process, and $\boldsymbol{\Gamma}(t)\boldsymbol{\Gamma}(t)^{\boldsymbol{T}} = \boldsymbol{\Sigma}(t)\boldsymbol{\Sigma}^{-1}\boldsymbol{\Sigma}(t)$. Therefore, for any $t \geq t_0 \geq 0$,

$$\hat{\boldsymbol{r}}(t)|\hat{\boldsymbol{r}}(t_0) = \hat{\boldsymbol{r}}(t_0) + \int_{t_0}^{t}\boldsymbol{\Gamma}(s)d\boldsymbol{B}_s.$$

For the more specialized setup with independent prior and likelihood, that is, $\boldsymbol{\Sigma}_z = \sigma_z^2\boldsymbol{I}$ and $\boldsymbol{\Sigma} = \sigma^2\boldsymbol{I}$, the expected reward process is given by

$$\hat{r}_j(t)|\hat{r}_j(t_0) = \int_{t_0}^{t}\frac{\sigma^{-1}}{\sigma_z^{-2}+t\sigma^{-2}}dB_s,$$

for either option $j \in \{1,2\}$. The magnitude of the fraction decreases over time, which indicates which the variability of the expected reward estimate decreases over time. This is to be expected, as later evidence influences this estimate less than early evidence.

The optimal policy for single, isolated choices

The objective function and associated value function

Assuming that accumulating evidence for some time t comes at cost $-ct$ (negative, as written in terms of reward), the decision maker's aim for single, isolated choices is to follow a policy that maximizes the total reward. This total reward is the reward experienced for choosing a particular option minus this accumulation cost, that is $\langle r_j(T)\rangle - c\langle T\rangle$, with expectation over choices and accumulation times. Given some expected reward estimates $(\hat{r}_1, \hat{r}_2)$ at time t, define the value function $V(t, \hat{r}_1, \hat{r}_2)$ as being the total expected reward to be received after time t while following the optimal policy, not including the accumulation cost up to time t. Thus, this value function is given by

$$V(t, \hat{r}_1, \hat{r}_2) = \max_{T\geq t}\langle \max\{\hat{r}_1(T), \hat{r}_2(T)\} - c(T-t)|\hat{r}_{1,2}(t) = \hat{r}_{1,2}\rangle,$$

where T is a stopping time, and the expectation is over possible trajectories of the expected reward process $\hat{\boldsymbol{r}}(T)|\hat{\boldsymbol{r}}(t)$. By definition of the value function, the expected total reward at choice option onset is given by $V(0, \bar{z}_1, \bar{z}_2)$, where we have used the fact that, a priori, $\hat{\boldsymbol{r}}(0) = \bar{\boldsymbol{z}}$.

Deriving Bellman's equation from the value function

To derive Bellman's equation [1] from the above expression of the value function we use the property that the T that we maximize over is not a random variance, and that $\hat{\boldsymbol{r}}(T)|\hat{\boldsymbol{r}}(t)$ is Markov, such that we can split the definition of the value function into $T = t$ and $T \geq t + \delta t$ for very small δt. For $T = t$, there won't be any further accumulation of evidence, such that the corresponding value is given by $\max\{\hat{r}_1, \hat{r}_2\}$. For $T \geq t + \delta t$, we are guaranteed to accumulate evidence for some duration δt at cost $-c\delta t$, after which the value corresponds to the maximization

$$\max_{T^* \geq t+\delta t} \left\langle \langle \max\{\hat{r}_1(T^*), \hat{r}_2(T^*)\} - c(T^* - (t+\delta t)) \,|\hat{r}_{1,2}(t+\delta t)\rangle \middle| \hat{r}_{1,2}(t) \right\rangle,$$

where the iterated expectation split the random process $\hat{r}_{1,2}(t) \to \hat{r}_{1,2}(T^*)$ into $\hat{r}_{1,2}(t) \to \hat{r}_{1,2}(t+\delta t) \to \hat{r}_{1,2}(T^*)$. The outer maximization is over the $\hat{r}_{1,2}(t+\delta t) \to \hat{r}_{1,2}(T^*)$, such that it can be moved into the outer expectation,

$$\left\langle \max_{T^* \geq t+\delta t} \langle \max\{\hat{r}_1(T^*), \hat{r}_2(T^*)\} - c(T^* - (t+\delta t)) \,|\hat{r}_{1,2}(t+\delta t)\rangle \,\middle| \hat{r}_{1,2}(t) \right\rangle.$$

By the definition of the value function, the above is equal to $\langle V(t+\delta t, \hat{r}_1(t+\delta t), \hat{r}_2(t+\delta t)) | \hat{r}_{1,2}(t)\rangle$. Returning to partitioning the value function into $T = t$ and $T \geq t + \delta t$, the function is given by the maximum over the value associated with either partition, resulting in Bellman's equation

$$V(t, \hat{r}_1, \hat{r}_2) = \max\left\{V_d(\hat{r}_1, \hat{r}_2), \langle V(t+\delta t, \hat{r}_1(t+\delta t), \hat{r}_2(t+\delta t)) | \hat{r}_{1,2}(t) = \hat{r}_{1,2}\rangle - c\delta t\right\},$$

where we have defined $V_d(\hat{r}_1, \hat{r}_2) = \max\{\hat{r}_1, \hat{r}_2\}$.

The value function is sub-linearly increasing in the expected rewards

Here, we derive some properties of the value function that will become useful to show that the optimal decision boundaries are parallel to the diagonal, as we will do in the next section. To derive these properties, we assume the evidence accumulation to be described by some stochastic process

$$\hat{\boldsymbol{r}}(t)|\hat{\boldsymbol{r}}(t_0) = \hat{\boldsymbol{r}}(t_0) + \int_{t_0}^{t} \boldsymbol{\mu}(s) ds + \int_{t_0}^{t} \boldsymbol{\Gamma}(s) d\boldsymbol{B}_s,$$

for some (possibly) time-dependent drift $\boldsymbol{\mu}(t)$ and (possibly) time-dependent diffusion covariance $\boldsymbol{\Gamma}(t)$. This process is more general than the one corresponding to the above task description by allowing a non-zero drift. Including such a non-zero drift is possible as it does not change the critical property of this process, which is that its evolution is invariant under the addition of a constant. That is, if we condition on $\hat{\boldsymbol{r}}^*(t_0) = \hat{\boldsymbol{r}}(t_0) + \boldsymbol{\Delta}_{\hat{\boldsymbol{r}}}$ instead of $\hat{\boldsymbol{r}}(t_0)$, where $\boldsymbol{\Delta}_{\hat{\boldsymbol{r}}}$ is some arbitrary two-dimensional vector, we have $\hat{\boldsymbol{r}}(t)|\hat{\boldsymbol{r}}^*(t_0) = (\hat{\boldsymbol{r}}(t)|\hat{\boldsymbol{r}}(t_0)) + \boldsymbol{\Delta}_{\hat{\boldsymbol{r}}}$, corresponding to a time-independent shift of the process. This property would not hold if either the drift $\boldsymbol{\mu}(t, \hat{\boldsymbol{r}})$ or diffusion covariance $\boldsymbol{\Gamma}(t, \hat{\boldsymbol{r}})$ were dependent on the current expected reward estimate. In what follows, we split the two-dimensional process into its components,

$$\hat{r}_1(t)|\hat{r}_1(t_0) = \hat{r}_1(t_0) + \int_{t_0}^{t} \mu_1(s) ds + \int_{t_0}^{t} \sigma_1(s)\sqrt{1-\rho(s)}\, dB_{1,s} + \int_{t_0}^{t} \sigma_1(s)\sqrt{\rho(s)} dB_s\,,$$
$$\hat{r}_2(t)|\hat{r}_2(t_0) = \hat{r}_2(t_0) + \int_{t_0}^{t} \mu_2(s) ds + \int_{t_0}^{t} \sigma_2(s)\sqrt{1-\rho(s)}\, dB_{2,s} + \int_{t_0}^{t} \sigma_1(s)\nu(t)\sqrt{\rho(s)} dB_s.$$

In the above, $\mu_1(t)$ and $\mu_2(t)$ are the drift terms, $\sigma_1^2(t)$ and $\sigma_2^2(t)$ are the diffusion variances, and $\rho(t) \in [0,1]$ and $\nu(t) \in \{-1,1\}$ determine magnitude and sign of the correlation between $\hat{r}_1$ and $\hat{r}_2$.

In what follows we demonstrate that, for any fixed $t \geq 0$,

- the value function is invariant under the addition of a constant, that is $V(t,\hat{r}_1,\hat{r}_2) + \Delta_{\hat{r}} = V(t,\hat{r}_1 + \Delta_{\hat{r}},\hat{r}_2 + \Delta_{\hat{r}})$ for any scalar $\Delta_{\hat{r}}$, that
- the value function is increasing in both $\hat{r}_1$ and $\hat{r}_2$, and that
- this increase is sub-linear, that is $V(t,\hat{r}_1 + \Delta_{\hat{r}},\hat{r}_2) \le V(t,\hat{r}_1,\hat{r}_2) + \Delta_{\hat{r}}$ and $V(t,\hat{r}_1,\hat{r}_2 + \Delta_{\hat{r}}) \le V(t,\hat{r}_1,\hat{r}_2) + \Delta_{\hat{r}}$.

The first two properties have already been demonstrated in [2] for a more restrictive expected reward process, but using the same approach as we are using here.

Invariance under addition of a constant

To demonstrate shift-invariance, fix some times $T_1 \ge t$ and $T_2 \ge t$ at which options 1 and 2 are chosen. For such decision times, $V(t,\hat{r}_1 + \Delta_{\hat{r}},\hat{r}_2 + \Delta_{\hat{r}})$ is given by

$$\begin{aligned}
&\langle\, 1_{T_1\le T_2}\hat{r}_1(T_1) + 1_{T_1>T_2}\hat{r}_2(T_2) - c(\min\{T_1,T_2\} - t) | \hat{r}_{1,2}(t) = \hat{r}_{1,2} + \Delta_{\hat{r}}\rangle \\
&= \langle\, 1_{T_1\le T_2}(\hat{r}_1(T_1) + \Delta_{\hat{r}}) + 1_{T_1>T_2}(\hat{r}_2(T_2) + \Delta_{\hat{r}}) - c(\min\{T_1,T_2\} - t) | \hat{r}_{1,2}(t) = \hat{r}_{1,2}\rangle \\
&= \langle\, 1_{T_1\le T_2}\hat{r}_1(T_1) + 1_{T_1>T_2}\hat{r}_2(T_2) - c(\min\{T_1,T_2\} - t) | \hat{r}_{1,2}(t) = \hat{r}_{1,2}\rangle + \Delta_{\hat{r}}
\end{aligned}$$

where $1_a = 1$ if a is true, and 0 otherwise, and was used to handle both $T_1 \le T_2$ (choosing option 1) and $T_1 > T_2$ (choosing option 2) simultaneously. Here, the first equality results from $\hat{\boldsymbol{r}}(T)|(\hat{\boldsymbol{r}}(t) + \Delta_{\hat{r}}\mathbf{1}) = (\hat{\boldsymbol{r}}(T)|\hat{\boldsymbol{r}}(t)) + \Delta_{\hat{r}}\mathbf{1}$, which is the essential property of our expected reward process, and the second equality results from $T_1 \le T_2$ and $T_1 > T_2$ being mutually exclusive events. The above holds for all choices of $T_1 \ge t$ and $T_2 \ge t$, such that it also holds for the maximum over these decision times. Therefore, $V(t,\hat{r}_1,\hat{r}_2) + \Delta_{\hat{r}} = V(t,\hat{r}_1 + \Delta_{\hat{r}},\hat{r}_2 + \Delta_{\hat{r}})$ for any scalar $\Delta_{\hat{r}}$.

Increasing in $\hat{r}_1$ and $\hat{r}_2$

A similar argument shows that the value function is increasing in both $\hat{r}_1$ and $\hat{r}_2$. Fix again some times $T_1 \ge t$ and $T_2 \ge t$ at which options 1 and 2 are chosen, such that $V(t,\hat{r}_1,\hat{r}_2)$ is given by

$$\begin{aligned}
&\langle\, 1_{T_1\le T_2}\hat{r}_1(T_1) + 1_{T_1>T_2}\hat{r}_2(T_2) - c(\min\{T_1,T_2\} - t) | \hat{r}_{1,2}(t) = \hat{r}_{1,2}\rangle \\
&= 1_{T_1\le T_2}\hat{r}_1 + \left\langle \left. \begin{matrix} 1_{T_1\le T_2}\left(\begin{matrix} \int_t^{T_1} \mu_1(s)ds + \int_t^{T_1} \sigma_1(s)\sqrt{1-\rho(s)}\, dB_{1,s} \\ + \int_t^{T_1} \sigma_1(s)\sqrt{\rho(s)} dB_s \end{matrix} \right) \\ + 1_{T_1>T_2}\hat{r}_2(T_2) - c(\min\{T_1,T_2\} - t) \end{matrix} \right| \hat{r}_{1,2}(t) = \hat{r}_{1,2} \right\rangle
\end{aligned}$$

where the second line results from substituting $\hat{r}_1(T_1)|\hat{r}_{1,2}(t) = \hat{r}_{1,2}$ by its stochastic process, and separating out $\hat{r}_1$. As $1_{T_1\le T_2} \ge 0$, the resulting expression is increasing in $\hat{r}_1$. This holds for any choice of $T_1 \ge t$ and $T_2 \ge t$, such that it also holds for $V(t,\hat{r}_1,\hat{r}_2)$. An analogue argument shows that the same holds for $\hat{r}_2$.

Increase is sub-linear

To show that the value function is sub-linearly increasing, fix again some decision times $T_1 \ge t$ and $T_2 \ge t$, with which, $V(t,\hat{r}_1 + \Delta_{\hat{r}},\hat{r}_2)$ can be written as

$$\begin{aligned}
&\langle\, 1_{T_1\le T_2}\hat{r}_1(T_1) + 1_{T_1>T_2}\hat{r}_2(T_2) - c(\min\{T_1,T_2\} - t) | \hat{r}_1(t) = \hat{r}_1 + \Delta_{\hat{r}}, \hat{r}_2(t) = \hat{r}_2\rangle \\
&= \langle\, 1_{T_1\le T_2}(\hat{r}_1(T_1) + \Delta_{\hat{r}}) + 1_{T_1>T_2}\hat{r}_2(T_2) - c(\min\{T_1,T_2\} - t) | \hat{r}_{1,2}(t) = \hat{r}_{1,2}\rangle \\
&= \langle\, 1_{T_1\le T_2}\hat{r}_1(T_1) + 1_{T_1>T_2}\hat{r}_2(T_2) - c(\min\{T_1,T_2\} - t) | \hat{r}_{1,2}(t) = \hat{r}_{1,2}\rangle + 1_{T_1\le T_2}\Delta_{\hat{r}} \\
&\le \langle\, 1_{T_1\le T_2}\hat{r}_1(T_1) + 1_{T_1>T_2}\hat{r}_2(T_2) - c(\min\{T_1,T_2\} - t) | \hat{r}_{1,2}(t) = \hat{r}_{1,2}\rangle + \Delta_{\hat{r}},
\end{aligned}$$

where the last inequality follows from $1_{T_1\le T_2} \le 1$. This holds for any choice of $T_1 \ge t$ and $T_2 \ge t$, such that $V(t,\hat{r}_1 + \Delta_{\hat{r}},\hat{r}_2) \le V(t,\hat{r}_1,\hat{r}_2) + \Delta_{\hat{r}}$. An analogous argument shows that $V(t,\hat{r}_1,\hat{r}_2 + \Delta_{\hat{r}}) \le V(t,\hat{r}_1,\hat{r}_2) + \Delta_{\hat{r}}$.

Note that, as soon as drift or diffusion of the expected reward process are dependent on the current expected reward estimates, none of the above properties can be guaranteed to hold in general.

The optimal decision boundaries are parallel to the diagonal

Equipped with these value function properties, we can demonstrate that the optimal decision boundaries are parallel to the diagonal. We will do so in two steps. First, we fix some t and $\hat{r}_1$ and show that for all $\hat{r}_2 \in [\xi_1(t,\hat{r}_1), \xi_2(t,\hat{r}_2)]$ within boundaries

$$\xi_1(t,\hat{r}_1) = \max\{\hat{r}_2 \leq \hat{r}_1 : V(t,\hat{r}_1,\hat{r}_2) = \hat{r}_1\},$$
$$\xi_2(t,\hat{r}_2) = \min\{\hat{r}_2 > \hat{r}_1 : V(t,\hat{r}_1,\hat{r}_2) = \hat{r}_2\},$$

it is optimal to accumulate more evidence, whereas outside of these boundaries it is better to choose option one (two) if $\hat{r}_2 \leq \xi_1(t,\hat{r}_1)$ ($\hat{r}_2 \geq \xi_2(t,\hat{r}_1)$). Second, we show that both boundaries satisfy $\xi_j(t,\hat{r}_1 + \Delta_{\hat{r}}) = \xi_j(t,\hat{r}_1) + \Delta_{\hat{r}}$, which makes them parallel to the diagonal $\hat{r}_1 = \hat{r}_2$. In our argument, we have chosen to bound $\hat{r}_2$ for some fixed t and $\hat{r}_1$. This is an arbitrary choice, and we could establish the same facts by an analogous argument that bounds $\hat{r}_1$ for some fixed t and $\hat{r}_2$ instead.

The optimal decision boundaries

To determine the optimal decision boundaries, $\xi_1(t,\hat{r}_1)$ and $\xi_2(t,\hat{r}_1)$ on $\hat{r}_2$, first observe that, by the definition of the value function, $V(t,\hat{r}_1,\hat{r}_2) \geq \max\{\hat{r}_1,\hat{r}_2\}$. Furthermore, as long as $V(t,\hat{r}_1,\hat{r}_2) > \max\{\hat{r}_1,\hat{r}_2\}$ it is best to accumulate more evidence, and to decide as soon as $V(t,\hat{r}_1,\hat{r}_2) = \max\{\hat{r}_1,\hat{r}_2\}$. Therefore, for any combination of $\hat{r}_1$ and $\hat{r}_2$, decisions ought to be made at the smallest t at which $V(t,\hat{r}_1,\hat{r}_2) = \max\{\hat{r}_1,\hat{r}_2\}$ holds. This will form the basis for finding the optimal decision boundaries.

Assume first that $\hat{r}_1 \geq \hat{r}_2$, in which case $\max\{\hat{r}_1,\hat{r}_2\} = \hat{r}_1$, such that $V(t,\hat{r}_1,\hat{r}_2) \geq \hat{r}_1$, and choosing option 1 is optimal as soon as $V(t,\hat{r}_1,\hat{r}_2) = \hat{r}_1$. The value function is increasing in $\hat{r}_2$, such that for any fixed t and $\hat{r}_1$, there exists some threshold $\xi_1(t,\hat{r}_1)$ in $\hat{r}_2$ below which $V(t,\hat{r}_1,\hat{r}_2) = \hat{r}_1$ for all $\hat{r}_2$ (as $V(t,\hat{r}_1,\hat{r}_2)$ cannot be smaller than $\hat{r}_1$). Above this threshold, we have $V(t,\hat{r}_1,\hat{r}_2) > \hat{r}_1$. Therefore, the threshold determines the optimal decision boundary for option 1, and is positioned at the largest $\hat{r}_2$ at which $V(t,\hat{r}_1,\hat{r}_2) = \hat{r}_1$ still holds, that is $\xi_1(t,\hat{r}_1) = \max\{\hat{r}_2 \leq \hat{r}_1 : V(t,\hat{r}_1,\hat{r}_2) = \hat{r}_1\}$.

Alternatively, we have $\hat{r}_1 < \hat{r}_2$, in which case $\max\{\hat{r}_1,\hat{r}_2\} = \hat{r}_2$, such that $V(t,\hat{r}_1,\hat{r}_2) \geq \hat{r}_2$, and choosing option 2 is optimal as soon as $V(t,\hat{r}_1,\hat{r}_2) = \hat{r}_2$. In this case, for any fixed t and $\hat{r}_1$, the value function is sub-linearly increasing in $\hat{r}_2$, such that it grows at most as fast as $\hat{r}_2$. Therefore, we will have $V(t,\hat{r}_1,\hat{r}_2) > \hat{r}_2$ for smaller $\hat{r}_2$, which will approach $V(t,\hat{r}_1,\hat{r}_2) = \hat{r}_2$ with growing $\hat{r}_2$ at some threshold $\xi_2(t,\hat{r}_1)$ in $\hat{r}_2$. This threshold determines the optimal decision boundary for option 2, and is positioned at the smallest $\hat{r}_2$ at which $V(t,\hat{r}_1,\hat{r}_2) = \hat{r}_2$ still holds, that is $\xi_2(t,\hat{r}_1) = \min\{\hat{r}_2 > \hat{r}_1 : V(t,\hat{r}_1,\hat{r}_2) = \hat{r}_2\}$.

The optimal decision boundaries are parallel to the diagonal

As the last step we show that both decision boundaries are parallel to the diagonal. For the first boundary, we have for some arbitrary scalar $\Delta_{\hat{r}}$,

$$\begin{aligned}
\xi_1(t,\hat{r}_1) + \Delta_{\hat{r}} &= \max\{\hat{r}_2 \leq \hat{r}_1 : V(t,\hat{r}_1,\hat{r}_2) = \hat{r}_1\} + \Delta_{\hat{r}} \\
&= \max\{\hat{r}_2^* \leq \hat{r}_1^* : V(t,\hat{r}_1^* - \Delta_{\hat{r}},\hat{r}_2^* - \Delta_{\hat{r}}) = \hat{r}_1^* - \Delta_{\hat{r}}\} \\
&= \max\{\hat{r}_2^* \leq \hat{r}_1^* : V(t,\hat{r}_1^*,\hat{r}_2^*) = \hat{r}_1^*\} \\
&= \xi_1(t,\hat{r}_1^*) \\
&= \xi_1(t,\hat{r}_1 + \Delta_{\hat{r}}),
\end{aligned}$$

where we have defined $\hat{r}_j^* = \hat{r}_j + \Delta_{\hat{r}}$ for both $j \in \{1,2\}$, and the third equality follows from the shift-invariance of the value function. The same argument applied to

$\xi_2(t,\hat{r}_1)+\Delta_{\hat{r}}$ results in $\xi_2(t,\hat{r}_1)+\Delta_{\hat{r}}=\xi_2(t,\hat{r}_1+\Delta_{\hat{r}})$. Therefore, both decision boundaries are parallel to the diagonal $\hat{r}_1=\hat{r}_2$.

The decision boundaries are independent of shifts of the prior

Assume prior means $\bar{z}_j^*=\bar{z}_j+\Delta_{\bar{z}}$, shifted for both options equally by some scalar $\Delta_{\bar{z}}$. From the shift invariance of the expected reward process we know that it will only affect the reward expectation estimates by a similar shift, that is $\hat{\boldsymbol{r}}(t)|(\hat{\boldsymbol{r}}(0)=\bar{\boldsymbol{z}}^*)=\left(\hat{\boldsymbol{r}}(t)|(\hat{\boldsymbol{r}}(0)=\bar{\boldsymbol{z}})\right)+\mathbf{1}\Delta_{\bar{z}}$. Furthermore, the shape of the value function is invariant to such shifts, as, by $V(t,\hat{r}_1+\Delta_{\bar{z}},\hat{r}_2+\Delta_{\bar{z}})=V(t,\hat{r}_1,\hat{r}_2)+\Delta_{\bar{z}}$, these shifts only change the value function's magnitude, but not its shape. As it is the value function's shape that determines the optimal decision boundaries, these decision boundaries are not affected by such shifts in the prior means.

Optimal decision boundaries might be non-parallel for non-linear utility functions

Here we show that a non-linear *utility function* u between experienced and true reward, $r_j=u(z_j)$ will cause the expected reward process to have a drift and/or diffusion that might depends on the current estimate of the expected rewards. As a consequence, the value function might cease to be invariant under addition of a constant, which might lead to non-parallel decision boundaries. Here, we only provide a theoretical argument. In the main text, we numerically compute the optimal decision boundaries for $u(z)=\tanh(z)$, and demonstrate that they are indeed non-parallel.

Our argument is based on the task setup with independent prior and likelihood, that is, for which $\boldsymbol{\Sigma}_z=\sigma_z^2\boldsymbol{I}$ and $\boldsymbol{\Sigma}=\sigma^2\boldsymbol{I}$. In this case, the posterior over the true value given evidence $\delta\boldsymbol{x}(0{:}\,t)$ is independent across choice options, and is for option j given by

$$z_j|\delta x_j(0{:}\,t)\sim\mathcal{N}\left(\frac{\sigma_z^{-2}\bar{z}_j+\sigma^{-2}x_j(t)}{\sigma_z^{-2}+t\sigma^{-2}},\frac{1}{\sigma_z^{-2}+t\sigma^{-2}}\right).$$

Given this posterior, our aim is to derive the process that describes the evolution of the expected reward estimate $\hat{r}_j(t)\equiv\langle u(z_j)|\delta x_j(0{:}\,t)\rangle$ over time. Given that t and $x_j(t)$ are sufficient statistics of the posterior z_j, the expected reward estimate will be fully determined by these statistics, which implies that there exists a function f such that $\hat{r}_j(t)=f(t,x_j)$. In the following, we first derive the expected reward process for some general f, and then will give its expression resulting from a linear and a non-linear $u(z)$.

To find the process for a general f, we assume f to be twice differentiable and invertible in x_j. By definition of the momentary evidence, we have $dx_j=z_jdt+\sigma dB_t$. Therefore, by Itō's Lemma,

$$df(t,x_j)=\left(\frac{\partial f}{\partial t}+z_j\frac{\partial f}{\partial x_j}+\frac{\sigma^2}{2}\frac{\partial^2 f}{\partial x_j^2}\right)dt+\sigma\frac{\partial f}{\partial x_j}dB_t$$

.

Using $\hat{r}_j(t)=f(t,x_j)$, and chopping the process into small time bins of size δt, the above results in

$$\hat{r}_j(t+\delta t)=\hat{r}_j(t)+\left(\frac{\partial f}{\partial t}+z_j\frac{\partial f}{\partial x_j}+\frac{\sigma^2}{2}\frac{\partial^2 f}{\partial x_j^2}\right)\delta t+\sigma\frac{\partial f}{\partial x_j}\sqrt{\delta t}\eta_t,$$

where $\eta_t\sim\mathcal{N}(0,1)$. Using this expression and marginalizing over z_j using the above posterior gives

$$\hat{r}_j(t+\delta t)|\hat{r}_j(t), x_j(t)$$
$$\sim \mathcal{N}\left(\hat{r}_j(t) + \left(\frac{\partial f}{\partial t} + \frac{\sigma_z^{-2}\bar{z}_j + \sigma^{-2}x_j(t)}{\sigma_z^{-2} + t\sigma^{-2}}\frac{\partial f}{\partial x_j} + \frac{\sigma^2}{2}\frac{\partial^2 f}{\partial x_j^2}\right)\delta t, \sigma^2\left(\frac{\partial f}{\partial x_j}\right)^2 \delta t\right),$$

such that the process governing $\hat{r}_j(t)$ is given by

$$d\hat{r}_j(t) = \left(\frac{\partial f}{\partial t} + \frac{\sigma_z^{-2}\bar{z}_j + \sigma^{-2}x_j(t)}{\sigma_z^{-2} + t\sigma^{-2}}\frac{\partial f}{\partial x_j} + \frac{\sigma^2}{2}\frac{\partial^2 f}{\partial x_j^2}\right)dt + \sigma\frac{\partial f}{\partial x_j}dB_t.$$

The above expression $x_j(t)$ still appears on the right-hand side, but its occurrences can be replace by $x_j(t) = f^{-1}\left(t, \hat{r}_j(t)\right)$ as f is invertible in the second argument.

A linear utility function

Let us turn to specific functions u. We first assume u to be linear, that is, $u(z) = az + b$. In this case, we find f to be given by

$$f(t, x_j) = \langle u(z_j)|\delta x_j(0\colon t)\rangle = a\frac{\sigma_z^{-2}\bar{z}_j + \sigma^{-2}x_j}{\sigma_z^{-2} + t\,\sigma^{-2}} + b.$$

Computing its derivatives with respect to t and x_j, and plugging them into the above expression for $d\hat{r}_j(t)$ results after a few lines of algebra in

$$d\hat{r}_j(t) = a\frac{\sigma^{-1}}{\sigma_z^{-2} + t\sigma^{-2}}dB_t,$$

which has no drift, and a diffusion that only depends on time. Therefore, a linear mapping between experienced and true reward results again in decision boundaries that are parallel to the diagonal.

A non-linear utility function

As an example of a non-linear u we use $u(z) = (1 - e^{-\sigma z})\sigma^{-1}$. This function increases non-linearly with a diminishing gradient to its asymptote σ^{-1}. We use this function rather than the $u(z) = \tanh(z)$ used for simulations in the main text, as it result in a closed-form f, given by

$$f(t, x_j) = \frac{1}{\sigma}\left(1 - e^{\frac{\frac{\sigma^2}{2} - \sigma\bar{z}_j\sigma_z^{-2} - \sigma^{-1}x_j}{\sigma_z^{-2} + t\sigma^{-2}}}\right).$$

Taking derivatives and substituting them into the above expression for $d\hat{r}_j(t)$ results after some lines of algebra and a fair amount of cancellations in

$$d\hat{r}_j(t) = \frac{\sigma^{-1} - \hat{r}_j(t)}{\sigma_z^{-2} + t\sigma^{-2}}dB_t.$$

In contrast to the linear case, the diffusion now depends on the current expected reward estimate $\hat{r}_j(t)$, such that the process ceases to remain invariant under the addition of a constant. This dependency on $\hat{r}_j(t)$ is required, as it ensures that $\hat{r}_j(t)$ remains upper-bounded by σ^{-1}, as imposed by our choice for u. As a result, we cannot guarantee anymore that the optimal decision boundaries remain parallel to the diagonal.

The optimal policy that maximizes the reward rate

The objective function and associated value function

In contrast to maximizing the total expected reward for a single, isolated choice, we now move to maximizing the total expected reward for an arbitrary number of choices within a temporally bounded interval. As we describe in the main text, as long as this interval is large enough, the objective becomes equivalent to maximizing the reward rate

$$\rho = \frac{\langle r_j | \delta \boldsymbol{x}(0{:}T) \rangle - c\langle T \rangle}{t_w + \langle T \rangle},$$

where the expectation is over choices and decision times, and t_w is the (average) waiting time between a choice and the next choice onset.

To find the optimal policy for this case, we move from using the standard value function $V(\cdot)$ to the average-adjusted value function $\tilde{V}(\cdot)$ that penalizes the passage of some time δt by the cost $-\rho \delta t$ [3,4]. As shown in **Methods** in the main text, this causes Bellman's equation to be given by

$$\tilde{V}(t, \hat{r}_1, \hat{r}_2, \rho) = \max \left\{ \begin{matrix} \tilde{V}_d(\hat{r}_1, \hat{r}_2, \rho), \\ \langle \tilde{V}(t + \delta t, \hat{r}_1(t + \delta t), \hat{r}_2(t + \delta t), \rho) | \hat{r}_{1,2}(t) = \hat{r}_{1,2} \rangle - (c + \rho)\, \delta t \end{matrix} \right\},$$

with $\tilde{V}_d(\hat{r}_1, \hat{r}_2, \rho) = \max\{\hat{r}_1, \hat{r}_2\} - \rho t_w$. The above is subject to the constraint $\tilde{V}(0, \bar{z}_1, \bar{z}_2, \rho) = 0$, which allows us to infer the value of ρ. This recursive definition of the value function can, as before, be written in a non-recursive way, given by

$$\tilde{V}(t, \hat{r}_1, \hat{r}_2, \rho) = \max_{T \geq t} \left\langle \max\{\hat{r}_1(T), \hat{r}_2(T)\} - (c + \rho)(T - t) \,\middle|\, \hat{r}_{1,2}(t) = \hat{r}_{1,2} \right\rangle - \rho t_w,$$

again subject to $\tilde{V}(0, \bar{z}_1, \bar{z}_2, \rho) = 0$. These two forms can be shown to be equivalent as before, by splitting the non-recursive form into a part corresponding to immediate decisions, $\max\{\hat{r}_1, \hat{r}_2\} - \rho t_w$, and one corresponding to accumulating evidence some more time δt and deciding later, $\left\langle \tilde{V}(t + \delta t, \hat{r}_1(t + \delta t), \hat{r}_2(t + \delta t), \rho) \middle| \hat{r}_{1,2}(t) \right\rangle - (c + \rho)\delta t$. Taking the maximum over these two parts results in Bellman's equation.

The optimal decision boundaries are parallel to the diagonal

The argument showing that the optimal decision boundaries are parallel to the diagonal for single, isolated trials relies on the three properties of the value function that we have derived further above. As long as these properties also hold for the average-adjusted value function, the optimal decision boundaries are again guaranteed to be parallel to the diagonal.

To show that this is indeed the case, consider the non-recursive form of the average-adjusted value function. This form differs from the value function for single, isolated trials in two points. First, the accumulation cost increases from $-c$ to $-(c + \rho)$. Second, an additional, final cost of $-\rho t_w$ is introduced. It is critical that neither of these changes influence the desired value function properties, which is easy to check by applying the same arguments we have used to demonstrate properties of the value function to the average-adjusted value function. This implies that the average-adjusted value function is invariant under the addition of a constant, is increasing in $\hat{r}_1$ and $\hat{r}_2$, and does so sub-linearly. These properties are the only ones required to show that, as for single, isolated choices, the optimal decision boundaries are again parallel to the diagonal.

The value function is strictly decreasing in the reward rate

Here we show that, without constraining the reward rate to satisfy $\tilde{V}(0, \bar{z}_1, \bar{z}_2, \rho) = 0$, this value function is strictly decreasing in the reward rate as long as $t_w > 0$ (rather

than $t_w = 0$). To do so, fix some times $T_1 \geq t$ and $T_2 \geq t$ at which options 1 and 2 are chosen. With these times fixed, the average-adjusted value function $\tilde{V}(t, \hat{r}_1, \hat{r}_2, \rho)$ can be written as

$$\left\langle 1_{T_1 \leq T_2} \hat{r}_1(T_1) + 1_{T_1 > T_2} \hat{r}_2(T_2) - c(\min\{T_1, T_2\} - t) \middle| \hat{r}_{1,2}(t) = \hat{r}_{1,2} \right\rangle - \rho(\min\{T_1, T_2\} - t + t_w).$$

As $T_1 \geq t$ and $T_2 \geq t$ we have $\min\{T_1, T_2\} - t \geq 0$. Therefore, $\min\{T_1, T_2\} - t + t_w > 0$, such that the above is strictly decreasing in ρ. This holds for any valid choice of T_1 and T_2, such that it also holds for the average-adjusted value function $\tilde{V}(t, \hat{r}_1, \hat{r}_2, \rho)$. If $t_w = 0$, the value function is still decreasing in ρ, but not necessarily strictly.

The reward rate is strictly increasing with positive shifts of the prior means

Assume $t_w > 0$, priors means $\bar{\mathbf{z}}^* = \bar{\mathbf{z}} + \Delta_{\bar{z}} \mathbf{1}$ for some scalar $\Delta_{\bar{z}}$, and reward rate ρ corresponding to prior means $\bar{\mathbf{z}}$ such that $\tilde{V}(0, \bar{z}_1, \bar{z}_2, \rho) = 0$. As the average-adjusted value function is invariant under the addition of a constant, we have $\tilde{V}(0, \bar{z}_1^*, \bar{z}_2^*, \rho) = \tilde{V}(0, \bar{z}_1 + \Delta_{\bar{z}}, \bar{z}_2 + \Delta_{\bar{z}}, \rho) = \tilde{V}(0, \bar{z}_1, \bar{z}_2, \rho) + \Delta_{\bar{z}} = \Delta_{\bar{z}}$. Assuming $\Delta_{\bar{z}} > 0$ (or $\Delta_{\bar{z}} < 0$), there exists some $\rho^* > \rho$ (or $\rho^* < \rho$) such that $\tilde{V}(0, \bar{z}_1^*, \bar{z}_2^*, \rho^*) = 0$, as the value function is strictly decreasing in ρ. Therefore, the reward rate is strictly increasing in $\Delta_{\bar{z}}$. If $t_w = 0$, the reward rate is still increasing in $\Delta_{\bar{z}}$, but not necessarily strictly.

Note that here we are ignoring a special boundary case: if the prior means become sufficiently negative and we have no accumulation cost, $c = 0$, then the chance of receiving positive reward for either choice becomes negligible. This means that making such choices will lead to a negative reward rate, whereas not choosing at all leads to a reward rate of zero. Thus, it is best to not choose at all. This will happen for all significantly negative prior means, such that for those, the reward rate will not be strictly increasing anymore. This also shows that, for $c = 0$, the reward rate is lower-bounded by zero. As soon as $c > 0$, it always becomes advantageous to make choices within some finite accumulation times, such that in these cases, the reward rate can become negative.

Larger prior means $\bar{z}$ imply faster choices

As we have shown in the last section, larger prior means imply higher reward rates. Here we show that such higher reward rates imply faster choices (or, more technically, never slower choices). To do so, assume prior means $\bar{\mathbf{z}}$ and $\bar{\mathbf{z}}^*$ (low and high) with associated reward rates ρ and ρ^*, such that $\rho^* = \rho + \Delta_\rho$ for some $\Delta_\rho > 0$. In what follows, we will show that if the optimal policy for ρ^* implies a choice at time T^* for expected reward estimates $\hat{r}_1$ and $\hat{r}_2$, then the optimal policy for ρ promotes accumulating more evidence for the same expected reward estimates. As a result, the policy associated with higher prior means leads to faster choices.

As a first step, we refine how the value function behaves with changes in the reward rate. Without considering $\tilde{V}(0, \bar{z}_1, \bar{z}_2, \rho) = 0$, fix some $T_1 \geq t$ and $T_2 \geq t$ at which options 1 and 2 are chosen. Then the average-adjusted value function $\tilde{V}\left(t, \hat{r}_1, \hat{r}_2, \rho + \Delta_\rho\right)$ for reward rate $\rho + \Delta_\rho$ and some $\Delta_\rho > 0$ can be written as

$$\begin{aligned} &\left\langle 1_{T_1 \leq T_2} \hat{r}_1(T_1) + 1_{T_1 > T_2} \hat{r}_2(T_2) - (c + \rho)(\min\{T_1, T_2\} - t) \middle| \hat{r}_{1,2}(t) = \hat{r}_{1,2} \right\rangle - \rho t_w \\ &\qquad - \Delta_\rho(\min\{T_1, T_2\} - t + t_w) \\ &\leq \left\langle 1_{T_1 \leq T_2} \hat{r}_1(T_1) + 1_{T_1 > T_2} \hat{r}_2(T_2) - (c + \rho)(\min\{T_1, T_2\} - t) \middle| \hat{r}_{1,2}(t) = \hat{r}_{1,2} \right\rangle - \rho t_w - \Delta_\rho t_w, \end{aligned}$$

where the inequality results from $\min\{T_1, T_2\} - t \geq 0$. The above holds for all valid T_1 and T_2, such that $\tilde{V}\left(t, \hat{r}_1, \hat{r}_2, \rho + \Delta_\rho\right) \leq \tilde{V}(t, \hat{r}_1, \hat{r}_2, \rho) - \Delta_\rho t_w$. This shows as before that the

value function is strictly decreasing in ρ (for $t_w > 0$), but now additionally provides the slope, $\frac{\partial \tilde{V}(t,\hat{r}_1,\hat{r}_2,\rho)}{\partial \rho} \leq -t_w$, for this decrease.

Let us now fix the expected reward estimates, $\hat{r}_1$ and $\hat{r}_2$, and consider at which time it were optimal to make a choice given that these are our current estimate. As we have discussed further above for single, isolated trials, the optimal decision time is the smallest time at which the value for deciding equals the value function, that is, where $V(t,\hat{r}_1,\hat{r}_2) = \max\{\hat{r}_1,\hat{r}_2\}$ holds. Adjusting to the reward rate case, we instead need $\tilde{V}(t,\hat{r}_1,\hat{r}_2,\rho) = \max\{\hat{r}_1,\hat{r}_2\} - \rho t_w$ to hold. Thus, for reward rate ρ^*, the optimal decision time is

$$T^* = \min\{t: \max\{\hat{r}_1,\hat{r}_2\} = \tilde{V}(t,\hat{r}_1,\hat{r}_2,\rho^*) + \rho^* t_w\}.$$

Observe that, due to $\rho^* = \rho + \Delta_\rho$, the left-hand side of the condition within brackets is lower-bounded by

$$\begin{aligned}\tilde{V}\left(T^*,\hat{r}_1,\hat{r}_2,\rho+\Delta_\rho\right) + \left(\rho+\Delta_\rho\right)t_w &\leq \tilde{V}\left(T^*,\hat{r}_1,\hat{r}_2,\Delta_\rho\right) - \Delta_\rho t_w + \left(\rho+\Delta_\rho\right)t_w \\ &= \tilde{V}(T^*,\hat{r}_1,\hat{r}_2,\rho) + \rho t_w,\end{aligned}$$

such that at the optimal decision time T^* associated with reward rate ρ^* we have $\max\{\hat{r}_1,\hat{r}_2\} - \rho t_w \leq \tilde{V}(T^*,\hat{r}_1,\hat{r}_2,\rho)$. This implies that, for reward rate ρ, the value for waiting might be higher than that for deciding, such that it might be better to accumulate more evidence. Thus, the optimal decision time associated with reward rate ρ, given by $T = \min\{t: \max\{\hat{r}_1,\hat{r}_2\} = \tilde{V}(T,\hat{r}_1,\hat{r}_2,\rho) + \rho t_w\}$, is at least as large as T^*. Therefore, decision times for the larger reward rates are shorter than (or, technically, at most as long as) those for smaller reward rates.

The policy that maximizes the correct rate

Evidence accumulation and expected rewards

We assume the same general evidence-generating process as in the previous sections, with prior $\boldsymbol{z} \sim \mathcal{N}(\bar{\boldsymbol{z}}, \boldsymbol{\Sigma}_z)$ and momentary evidence $\delta\boldsymbol{x}_i \sim \mathcal{N}(\boldsymbol{z}\delta t, \boldsymbol{\Sigma}\delta t)$. This results in the posterior $\boldsymbol{z}|\delta\boldsymbol{x}(0:t)$ as discussed at the beginning of this document, $\boldsymbol{z}|\delta\boldsymbol{x}(0:t) \sim \mathcal{N}(\hat{\boldsymbol{z}}(t), \boldsymbol{\Sigma}(t))$.

In contrast to the previous sections, we do equate experience reward $\boldsymbol{r}$ and true reward $\boldsymbol{z}$, but instead that the decision maker receives reward R_{corr} for choosing the option j associated with the larger z_j, and R_{incorr} otherwise. With the above posterior, we have for $j \neq i$,

$$p\left(z_j > z_i | t, \hat{z}_1, \hat{z}_2\right) = \int_{-\infty}^{0} \mathcal{N}\left(\Delta z_{ij} | \hat{z}_i - \hat{z}_j, \Delta\sigma^2(t)\right) d\Delta z_{ij} = \Phi\left(\frac{\hat{z}_j - \hat{z}_i}{\sqrt{\Delta\sigma^2(t)}}\right),$$

where, in the first equality we have defined $\Delta z_{ij} = z_i - z_j$ and $\Delta\sigma^2(t) = \Sigma_{11}(t) + \Sigma_{22}(t) - 2\,\Sigma_{12}(t)$ (with $\Sigma_{ij}(t)$ denoting components of $\boldsymbol{\Sigma}(t)$), and $\Phi(\cdot)$ in the last term is the cumulative distribution function for the standard Gaussian $\mathcal{N}(0,1)$. Therefore, the expected reward for choosing option j becomes

$$\begin{aligned}\langle r_j | \delta\boldsymbol{x}(0:t)\rangle &= R_{corr}\, p\left(z_j > z_i | t, \hat{z}_1, \hat{z}_2\right) + R_{incorr} p\left(z_i \geq z_j | t, \hat{z}_1, \hat{z}_2\right) \\ &= R_{corr}\Phi\left(\frac{\hat{z}_j - \hat{z}_i}{\sqrt{\Delta\sigma^2(t)}}\right) + R_{incorr}\Phi\left(\frac{\hat{z}_i - \hat{z}_j}{\sqrt{\Delta\sigma^2(t)}}\right).\end{aligned}$$

Importantly, the above is only a function of the difference of $\hat{z}_i$ and $\hat{z}_j$ rather than their individual values.

Bellman's equation for single choices and correct rate maximization

The statistics $(t, \hat{z}_1, \hat{z}_2)$ are sufficient for both the posterior $\boldsymbol{z}|\delta x(0:t)$ and the expected reward for choosing either option. Therefore, we can define the value function over these statistics. The work in Drugowitsch et al. (2011) [4], which is closely related to the setup considered in this section, used a slightly simpler evidence-generating process, which allowed the use of alternative sufficient statistics (t, g), where $g \equiv p(z_1 > z_2 | t, \hat{z}_1, \hat{z}_2)$.

Single, isolated choices

For single, isolated choices, the value for deciding immediately is the maximum over the expected rewards for either option, and is therefore given by

$$V_d(t, \hat{z}_1, \hat{z}_2) = \max \begin{Bmatrix} R_{corr} \Phi\left(\frac{\hat{z}_1 - \hat{z}_2}{\sqrt{\Delta\sigma^2(t)}}\right) + R_{incorr} \Phi\left(\frac{\hat{z}_2 - \hat{z}_1}{\sqrt{\Delta\sigma^2(t)}}\right), \\ R_{corr} \Phi\left(\frac{\hat{z}_2 - \hat{z}_1}{\sqrt{\Delta\sigma^2(t)}}\right) + R_{incorr} \Phi\left(\frac{\hat{z}_1 - \hat{z}_2}{\sqrt{\Delta\sigma^2(t)}}\right) \end{Bmatrix}.$$

The value for accumulating more evidence remains unchanged from before, and results in the value function

$$V(t, \hat{z}_1, \hat{z}_2) = \max_{T \geq t} \left\langle V_d(T, \hat{z}_1(T), \hat{z}_2(T)) - c(T - t) \middle| \hat{z}_{1,2}(t) = \hat{z}_{1,2} \right\rangle,$$

with associated Bellman Equation

$$V(t, \hat{z}_1, \hat{z}_2) = \max\left\{V_d(t, \hat{z}_1, \hat{z}_2), \left\langle V(t + \delta t, \hat{z}_1(t + \delta t), \hat{z}_2(t + \delta t)) \middle| \hat{z}_{1,2}(t) = \hat{z}_{1,2} \right\rangle - c\delta t\right\}.$$

In both cases, the expectation is over the temporal evolution of $\hat{\boldsymbol{z}}(t)$.

Correct rate maximization

For correct rate maximization, we again move to the average-adjusted value function, $\tilde{V}(t, \hat{z}_1, \hat{z}_2, \rho)$, where ρ is the correct rate. As before, the difference between the standard and the average-adjusted value function is that the latter additionally penalizes the passage of some time δt by $-\rho\delta t$. Fixing $\tilde{V}(0, \bar{z}_1, \bar{z}_2, \rho) = 0$, the average-adjusted value for deciding immediately is thus given by

$$\tilde{V}_d(t, \hat{z}_1, \hat{z}_2, \rho) = V_d(t, \hat{z}_1, \hat{z}_2) - \rho t_w.$$

Therefore, the overall adjusted value function is

$$\tilde{V}(t, \hat{z}_1, \hat{z}_2, \rho) = \max_{T \geq t} \left\langle \tilde{V}_d(T, \hat{z}_1(T), \hat{z}_2(T), \rho) - (c + \rho)(T - t) \middle| \hat{z}_{1,2}(t) = \hat{z}_{1,2} \right\rangle,$$

with associated Bellman Equation

$$\tilde{V}(t, \hat{z}_1, \hat{z}_2, \rho) = \max \begin{Bmatrix} \tilde{V}_d(t, \hat{z}_1, \hat{z}_2, \rho), \\ \left\langle \tilde{V}(t + \delta t, \hat{z}_1(t + \delta t), \hat{z}_2(t + \delta t), \rho) \middle| \hat{z}_{1,2}(t) = \hat{z}_{1,2} \right\rangle - (c + \rho)\delta t \end{Bmatrix}.$$

As before, the expectation is in both cases over the temporal evolution of $\hat{\boldsymbol{z}}(t)$.

The optimal policy is invariant to shifts in the prior mean

In contrast to the optimal policy associated with maximizing the reward rate, the optimal policy that maximizes the correct rate remains unchanged when adding the same constant to both elements of the prior mean $\bar{\mathbf{z}}$. This is shown in two steps. First, we show that introducing such a shift in the prior mean does not affect how the $\hat{\boldsymbol{z}}(t)$ evolves over time (other than being shifted as a whole). Second, we show that the value for deciding immediately is only sensitive to the difference $\hat{z}_1(t) - \hat{z}_2(t)$ rather than the individual values of these estimates. Together, this allows us to show that the value

function is unaffected by shifts in the prior mean, such that the associated optimal policy is neither.

We have previously shown that the process describing the evolution of the expected reward estimate, $\hat{\boldsymbol{r}}(t)$, features drift and diffusion that only depends on time, but not on its current estimate. This was sufficient such that the whole process could be shifted without affecting its evolution, that is $\hat{\boldsymbol{r}}(t)|(\hat{\boldsymbol{r}}(t_0) + \boldsymbol{\Delta}_{\hat{r}}) = (\hat{\boldsymbol{r}}(t)|\hat{\boldsymbol{r}}(t_0)) + \boldsymbol{\Delta}_{\hat{r}}$ for $t \geq t_0$ and some two-dimensional vector $\boldsymbol{\Delta}_{\hat{r}}$. This was demonstrated under the assumption that $\boldsymbol{r} = \boldsymbol{z}$, such that we have $\hat{\boldsymbol{z}}(t)|(\hat{\boldsymbol{z}}(t_0) + \boldsymbol{\Delta}_{\hat{z}}) = (\hat{\boldsymbol{z}}(t)|\hat{\boldsymbol{z}}(t_0)) + \boldsymbol{\Delta}_{\hat{z}}$ for the setup considered in this section. Therefore, the time evolution of $\hat{\boldsymbol{z}}(t)$, that is, how $\hat{\boldsymbol{z}}(t)$ changes in relative terms, is unaffected by a shift in the prior mean, as $\hat{\boldsymbol{z}}(t)|(\hat{\boldsymbol{z}}(0) = \bar{\boldsymbol{z}} + \boldsymbol{\Delta}_{\bar{z}}) = \left(\hat{\boldsymbol{z}}(t)|(\hat{\boldsymbol{z}}(0) = \bar{\boldsymbol{z}})\right) + \boldsymbol{\Delta}_{\bar{z}}$.

Furthermore, both $V_d(t, \hat{z}_1, \hat{z}_2)$ and its average-adjusted counterpart, $\tilde{V}_d(t, \hat{z}_1, \hat{z}_2, \rho)$, are only sensitive to $\hat{z}_1(t) - \hat{z}_2(t)$ rather than the individual values of these estimates. To see this, expand $V_d(t, \hat{z}_1 + \Delta_{\hat{z}}, \hat{z}_2 + \Delta_{\hat{z}})$ for any scalar $\Delta_{\hat{z}}$, which immediately results in $V_d(t, \hat{z}_1 + \Delta_{\hat{z}}, \hat{z}_2 + \Delta_{\hat{z}}) = V_d(t, \hat{z}_1, \hat{z}_2)$. The same procedure demonstrates $\tilde{V}_d(t, \hat{z}_1 + \Delta_{\hat{z}}, \hat{z}_2 + \Delta_{\hat{z}}, \rho) = \tilde{V}_d(t, \hat{z}_1, \hat{z}_2, \rho)$.

Both of these properties in combination imply that $V(t, \hat{z}_1 + \Delta_{\hat{z}}, \hat{z}_2 + \Delta_{\hat{z}}) = V(t, \hat{z}_1, \hat{z}_2)$. To see this, fix some $T_1 \geq t$ and $T_2 \geq t$ at which options 1 and 2 are chosen. Then, $V(t, \hat{z}_1 + \Delta_{\hat{z}}, \hat{z}_2 + \Delta_{\hat{z}})$ can be written as

$$
\begin{aligned}
&\left\langle V_d\left(T, \hat{z}_1(T), \hat{z}_2(T)\right) - c(T - t) \middle| T = \min\{T_1, T_2\}, \hat{z}_{1,2}(t) = \hat{z}_{1,2} + \Delta_{\hat{z}} \right\rangle \\
&= \langle V_d(T, \hat{z}_1(T) + \Delta_{\hat{z}}, \hat{z}_2(T) + \Delta_{\hat{z}}) - c(T - t) | T = \min\{T_1, T_2\}, \hat{z}_{1,2}(t) = \hat{z}_{1,2} \rangle \\
&= \left\langle V_d\left(T, \hat{z}_1(T), \hat{z}_2(T)\right) - c(T - t) \middle| T = \min\{T_1, T_2\}, \hat{z}_{1,2}(t) = \hat{z}_{1,2} \right\rangle,
\end{aligned}
$$

where the first equality is based on shifting $\hat{\boldsymbol{z}}(t)$, and the second equality uses the property of $V_d(\cdot)$ discussed above. As this holds for all valid decision times, T_1 and T_2, it also holds for $V(t, \hat{z}_1 + \Delta_{\hat{z}}, \hat{z}_2 + \Delta_{\hat{z}})$, such that $V(t, \hat{z}_1 + \Delta_{\hat{z}}, \hat{z}_2 + \Delta_{\hat{z}}) = V(t, \hat{z}_1, \hat{z}_2)$. A similar argument leads to $\tilde{V}(t, \hat{z}_1 + \Delta_{\hat{z}}, \hat{z}_2 + \Delta_{\hat{z}}, \rho) = \tilde{V}(t, \hat{z}_1, \hat{z}_2, \rho)$. Therefore, the optimal policy is the same for prior mean $\bar{\boldsymbol{z}}$ and $\bar{\boldsymbol{z}} + \Delta_{\bar{z}}\boldsymbol{1}$.

Supplementary Note 2

The sensitivity to the absolute reward magnitudes (**Fig. 5A**) is a remarkable property that differentiates value-based decision-making from classic perceptual decision-making. In a typical perceptual decision-making paradigm, the decision maker is rewarded based on whether the answer is "correct" or "incorrect," but not the values of options themselves. Therefore, the optimal strategy for perceptual decisions is to maximize the correct response rate. In value-based decisions, instead, subjects are always rewarded, even if they choose the "incorrect", lower-rewarding option (due to the stochastic realization of the evidence). This difference implies that a strategy that maximizes the correct-rate in value-based tasks does not necessarily maximize the reward-rate. Indeed, a strategy that maximizes the reward rate is sensitive to absolute reward magnitudes, unlike one that maximizes only the correct rate. In addition to the sensitivity to absolute reward magnitudes, the optimal boundaries in the value-based case tend to approach each other more rapidly (with a steeper slope over time) than for perceptual decisions (optimal asymptotic rate $1/t$ rather than $1/\sqrt{t}$, see [2,5,6]).

Supplementary Note 3

Oud et al.[7] recently demonstrated that humans can behave suboptimally in some forms of value-based decision-making tasks. Indeed, using a new stimulus-dependent reward design, they reported that human subjects tend to be slower than expected from a policy that maximizes reward rate for both value-based and perceptual decisions suggesting suboptimal decisions. Indeed reward rates could be increased by forcing subjects to wait shorter by imposing artificial deadlines. Although the exact source of this sub-optimality has yet to be clarified, there are multiple possibilities of how it could arise. In particular, our theoretical results demonstrate that prior knowledge of the reward distribution critically affects how the boundaries ought to collapse to maximize the reward rate. This implies that incorrectly or incompletely learned priors (or misunderstanding the task, e.g., using the accuracy-based strategy for value-based tasks) can result in exceedingly slow choices even when subjects follow the optimal policy for the prior they have learned.

There is already preliminary evidence that human subjects can adjust their decision policy in response to changes in the prior reward distribution. For instance, Otto and Daw (*Comput. Syst. Neurosci. Abstr.*, 2016) have found that subjects tend to respond faster in a value-based decision-making task following increases in reward rates, in line with our prediction. However, it is still unclear how long it takes subjects to fully adjust their policies in response to a change in the prior reward distribution. This question will require further experiments with long trial blocks using distinct task statistics (e.g., different expected rewards within each block).

Supplementary References

1. Bellman, R. E. *Dynamic Programming*. (1957).
2. Fudenberg, D., Strack, P. & Strazalecki, T. *Stochastic Choice and Optimal Sequential Sampling*. (2015). at <Available at SSRN: http://ssrn.com/abstract=2602927 or http://dx.doi.org/10.2139/ssrn.2602927>
3. Mahadevan, S. Average reward reinforcement learning: Foundations, algorithms, and empirical results. *Mach. Learn.* **22,** 159–195 (1996).
4. Drugowitsch, J., Moreno-Bote, R., Churchland, A. K., Shadlen, M. N. & Pouget, A. The cost of accumulating evidence in perceptual decision making. *J. Neurosci.* **32,** 3612–28 (2012).
5. Chernoff, H. Sequential tests for the mean of a normal distribution. *Proc. Fourth Berkeley Symp. Math. Stat. Probab.* **1,** 79–91 (1961).
6. Bather, J. A. Bayes procedures for deciding the sign of a normal mean. *Math. Proc. Cambridge Philos. Soc.* **58,** 599–620 (1962).
7. Oud, B. *et al.* Irrational time allocation in decision-making. *Proc. R. Soc. B Biol. Sci.* **283,** 20151439 (2016).
